# Naringenin impairs mitochondrial function via ROS to induce apoptosis in tamoxifen resistant MCF-7 breast cancer cells

**DOI:** 10.1371/journal.pone.0320020

**Published:** 2025-04-03

**Authors:** Lauren A. Eanes, Mayar Eldeeb, Darrell Storholt, Yashomati M. Patel

**Affiliations:** Department of Biology, University of North Carolina at Greensboro, Greensboro, North Carolina, United States of America; Sun Yat-Sen University, CHINA

## Abstract

Breast cancer is the second leading cause of cancer deaths among women. While tamoxifen, a commonly used drug therapy in breast cancer patients, is effective, many patients acquire tamoxifen resistance. Therefore, it is essential to identify alternative or combination therapeutics for the treatment of breast cancer. Naringenin, a naturally occurring flavonoid, has been reported to elicit antioxidant, anti-proliferative, and pro-apoptotic effects in cancer cells. The current study aimed to identify the mechanism by which naringenin induces apoptosis in tamoxifen-resistant breast cancer cells. The present study demonstrated that naringenin induced an increase in ROS, resulting in oxidative stress, impaired mitochondrial function, and apoptosis in tamoxifen-resistant breast cancer cells. Our study reports that naringenin specifically increases mitochondrial superoxide anions and hydrogen peroxide production while also causing mitochondrial dysfunction. These studies provide novel evidence for the mechanism by which naringenin induces apoptosis in tamoxifen-resistant breast cancer cells and supports the use of naringenin as a therapeutic on breast cancer cells and drug-resistant cancer cells.

## Introduction

Breast cancer is currently the most commonly diagnosed cancer in the world and is among the leading causes of cancer mortality in women worldwide [1]. Among women in the United States, breast cancer is second only to lung cancer. Breast cancer has the highest mortality and is a leading cause of cancer related deaths in women younger than forty [2]. Approximately 1 in 8 women are diagnosed with breast cancer in their lifetime [3]. Most breast cancers are hormone receptor-positive; thus, commonly used chemopreventatives and chemotherapies are selective estrogen receptor modulators (SERMS). A meta-analysis reported that SERMS reduced the risk of breast cancer by approximately 38% over ten years among high-risk women [4]. Tamoxifen, a SERM with minimal side effects, has been widely used for many years as a treatment of hormone receptor-positive breast cancers [5,6]. However, it has been reported that within five years of tamoxifen use, cancer cells can become resistant to the anti-estrogen effects of the drug, thus allowing the cells to bypass the estrogen-dependent pathways require for proliferation [7]. Thus, the need for alternative or complementary therapeutics to treat drug-resistant breast cancers is critical.

In a recent systematic review, the anti-cancer potential of naringenin was comprehensively examined [8]. Previous studies have highlighted the potential of naringenin, a flavanone present in citrus fruits, for its anti-cancer properties [9–15]. This compound has demonstrated the ability to inhibit the proliferation of cancer cells and induce apoptosis in various cancer cell types [9,14,16–18]. Our previous studies have demonstrated that chronic treatment (96 h) with naringenin (200-250 µM) promotes apoptosis and inhibits cell proliferation in MCF-7 breast cancer cells as well as tamoxifen-resistant (Tam-R) MCF-7 cells [19–21]. We have also shown that co-treatment with both naringenin and tamoxifen provides a more potent effect than either treatment alone in both MCF-7 [19] and Tam-R MCF-7 cells [20]. Specifically, naringenin has been shown to activate PARP, caspase 7, caspase 9, and to induce apoptosis by triggering the mitochondrial intrinsic pathway various cancer cell lines including MCF-7 cells [8,12,19,20]. These findings suggest that naringenin holds promise as a chemopreventive and chemotherapeutic agent [12,18,22]. However, what remains unknown is how naringenin triggers the apoptotic pathway.

Naringenin has been shown to regulate reactive oxygen species (ROS) levels, and increased levels of ROS are known to induce the apoptotic pathway. Regulation of ROS is currently gaining recognition as a promising target for cancer treatment [23]. Naringenin has been shown to function as antioxidant and free radical scavenger with minimal side effects in noncancer cells, however in cancer cells it has been shown to induce apoptosis by increasing ROS levels [8,24–29]. Although cancer has characteristically increased ROS generation, this increase is also balanced by an increase in antioxidant capabilities and is required to promote growth signaling pathways and DNA instability [30,31]. However, when ROS levels increase to a level to induce oxidative stress in cancer cells, this results in membrane damage, specifically in the mitochondria, causing leaky membranes and mitochondrial disfunction [32]. The mitochondria is a primary source of ROS and thus is vulnerable to damage during oxidative stress conditions, and these vulnerabilities have been linked to an adaptive response leading to drug resistance in cancer cells [32]. However, the mechanism by which naringenin promotes apoptosis and decreased proliferation in tamoxifen-resistant cancer cells remains unclear.

This study aimed to determine the mechanism by which naringenin induces apoptosis in Tam-R MCF-7 breast cancer cells. We show that naringenin promotes ROS production, specifically the production of mitochondrial associated superoxide which contributes to decreased mitochondrial membrane potential, and thus apoptosis. Our study demonstrated for the first time that a naringenin-induced increase in superoxide anions generated by the mitochondria causes a leaky mitochondrial membrane and, thus, a loss of membrane potential and mitochondrial function in Tam-R MCF-7 cells. Therefore, naringenin-induced apoptosis may result from oxidative stress and mitochondrial damage. The findings reported here suggest that the effects observed in naringenin-treated Tam-R breast cancer cells overcome the resistance-induced adaptation for growth and survival.

## Materials and methods

### Materials

MCF-7 ER + breast cancer cells (HTB-22) were purchased from ATCC. Dulbecco’s Modified Eagle Medium (DMEM) and penicillin-streptomycin (Pen-strep) were from Gibco. Fetal bovine serum (FBS), naringenin, 4-OH-tamoxifen, anti-mouse and anti-rabbit horseradish peroxidase conjugated secondary antibodies were obtained from Sigma Aldrich. Guava Nexin reagent and Guava Cell Cycle Reagent were purchased from Millipore. CM-H_2_DCFDA and MitoSOX were from ThermoFisher. The ATP detection kit was purchased from Abcam. The SYBR green Master Mix was from Life Technologies. The enhanced chemiluminescence (ECL) detection kit was from BioExpress.

### Cell culture

Tam-R cells were cultured in Dulbecco’s Modified Eagle Medium (DMEM) supplemented with 10% FBS, 0.01 mg/mL bovine insulin, and 100 U/mL penicillin/streptomycin and were maintained in 100 nM tamoxifen. Cells were incubated at 37°C and 5% CO_2_. Media was replaced every two days and cells were passaged at 80% confluency. Tam-R cells (2.45 ×  10^4^/plate) in growth phase were plated and either treated with DMSO (vehicle control) or 200 μM naringenin for various times as indicated.

### Annexin V-PE/7-AAD apoptosis detection

Apoptosis (Annexin V positive cells) was detected with Guava Nexin reagent for flow cytometry (Millipore 4500-0450) following the manufacturer’s protocol. Tam-R MCF-7 cells were treated with DMSO or 200 µM naringenin for 24, 48, or 96 h. The cells were then harvested, washed with 1x phosphate buffer saline (PBS) and stained with Annexin V-PE and 7-AAD respectively for 20 min following the manufacturer’s protocol. Fluorescence was analyzed by excitation/emission at 488/530nm on a GuavaCyte flow cytometer using NEXIN.

### Cell cycle analysis

Tam-R MCF-7 cells were harvested and re-suspended in 1 × PBS. Guava Cell-Cycle Reagent (Millipore 4500-0220) was added to cells at a 1:20 or 1:10 dilution and incubated for five min at room temperature in the dark. A GuavaCyte Flow Cytometry was used to count the number of cells within G_0_/G_1_, S, and M phases. Values were analyzed using GuavaSoft software to identify total cells within each phase of the cell cycle.

### Total reactive oxygen species (ROS) detection with CM-H_2_DCFDA

General ROS was measured using chloromethyl derivative of 2^’^7^’^-Dichlorofluorescein diacetate (CM-H_2_DCFDA) dye which passively diffuses into cells, binds with intracellular glutathione and other thiols and if oxidized, fluoresces. Tam-R MCF-7 cells were harvested and resuspended in Hank’s balanced salt solution (HBSS) with 1 µ M CM-H_2_DCFDA dye for 60 min followed by treatment with DMSO or 200 µ M naringenin for a 2, 3, or 6 h. Cells were washed with HBSS two times and resuspended in 1 ml PBS to measure hydrogen peroxide accumulation at excitation/emission of 495/529 nm using InCyte software on a GuavaCyte flow cytometer.

### MitoSOX red mitochondrial oxidative stress

For acute exposure to naringenin (200 µ M), Tam-R MCF-7 cells were harvested and resuspended in HBSS containing 5 µ M mitoSOX red dye for 30 min followed by treatment with DMSO or naringenin for 2, 3, or 6 h. For chronic exposure to naringenin (200 µ M), cells were pre-treated for 24, 48, or 96 h with DMSO or naringenin then harvested and incubated with mitoSOX for 30 min. Cells were washed with HBSS two times and resuspended in 1 ml of PBS to measure superoxide at excitation/emission of 510/580nm using InCyte software on a GuavaCyte Flow Cytometer.

### Quantitative real-time PCR (qRT-PCR)

Tam-R MCF-7 cells were treated chronically (48, and 96 h) with DMSO or 200 µ M naringenin. Total RNA was extracted using a Qiagen RNeasy mini prep kit. The purity and quantity of the isolated RNA were assessed. Power SYBR Green RNA-to-CT 1-Step Kit was used to reverse transcribe the RNA to cDNA and amplify target genes. The specific primers for GAPDH were forward 5’-CGACCACTTTGTCAAGCTCA-3’ and reverse 5’-AGGGGTCTACATGGCAACTG-3’; GPx1 forward 5’-TGGACCTTCAC CAGACCTAC-3’ and reverse 5’-GGTTGAGGAGAGAAACACCA-3’; Catalase forward 5’-TTCGGTTCTCCACTGTTGCT-3’ and reverse 5’-GATGTGTCTGAG GATTTCTCTTTTG-3’; SOD1 forward 5’-GCAATGTGACTGCTGACAAAGAT-3’ and reverse 5’-ATTACAC CACAAGCCAAACGACT-3’; SOD2 forward 5’-TGGACAAACCT CAGCCCTAAC-3’ and reverse 5’-GAAACCAAGCCAACCCCAAC-3’ (primers from personal communication with Dr. Zhenquan Jia at UNCG). The reaction cycles were performed in an Applied Biosystems thermal cycler. An initial 30 min hold at 48°C allowed for reverse transcription followed by a 10 min hold at 95°C to activate the DNA polymerase. A total of 40 cycles were performed for each run as follows: 95°C for 15 seconds, and 60°C for 60 sec followed by a melt curve cycle: 95°C for 15 sec, 60°C for 15 sec, and 95°C for 15 sec. Expression of the housekeeping gene, GAPDH, was also determined to normalize the expression of target genes. Quantification of gene expression was determined using the comparative threshold cycle CT method and expressed as fold-change compared to the untreated control.

### Immunoblotting

Protein extracts were subjected to 10% SDS-PAGE and then transferred to an Immobilon-P membrane. The membrane was incubated with the specific primary and secondary antibodies as indicated and visualized using enhanced chemiluminescence (ECL) and a Bio-Rad ChemiDoc XRS System. Protein bands were quantified using densitometric analysis using Quantity One analysis software.

### Mitochondrial membrane potential analysis

Tam-R MCF-7 cells were grown on coverslips for 24 h in DMEM media supplemented with DMSO or 200 µ M naringenin. Cells were stained for 30 min with 500 nM MitoTraker red at 37°C [33,34]. Following incubation cells were washed with 1xPBS (two times). Cells were fixed with 4% paraformaldehyde for 15 min and then washed twice with 1xPBS. Mitochondrial membrane potential was measured by fluorescence intensity using confocal microscopy. Intensity was quantified by measuring 20 cells per treatment for each treatment condition. Average fluorescence intensity was calculated and normalized to the untreated control.

### Statistical analysis

Data are presented as means ±  SEM. The significance of comparing means was assessed by two-way analysis of Student’s *t*-test.

## Results

### Naringenin impairs cell viability and induces apoptosis

**Fig 1:**
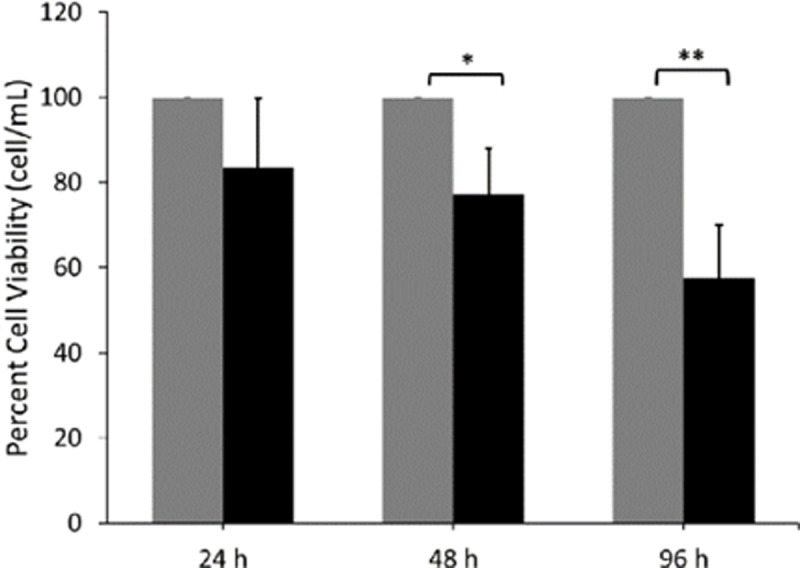
Naringenin decreases total cell viability. The Tam-R MCF-7 cells were treated for 24, 48, 96 h with DMSO (gray bar) or naringenin (200 µ M) (black bar). Cells were collected and incubated with Guava ViaCount reagent. Cells were analyzed for viability using ViaCount Software on Guave EasyCyte Flow Cytometer. The total viable cell count was normalized to total cell count within each treatment group was normalized to the total cell count within each treatment group and then normalized to the DMSO control level for each time point. The data are represented as a mean ± SEM of 5 independent trials. * p-value ≤  0.05 **p-value ≤  0.005.

While naringenin has been shown to induce apoptosis [9,14,19,20], the cellular mechanism is still unclear. In the present study, we aimed to determine the extent to which Tam-R cell viability was impaired by chronic naringenin treatment. Previous studies in our lab have demonstrated that a concentration of 100-200 µ M naringenin was optimal to impair cell proliferation and viability [8,19–21]. Tam-R cells were treated with either DMSO or naringenin for 24, 48, or 96 hours, and cell viability was determined. The total number of viable cells was normalized to the total cell count per treatment and then normalized to the DMSO-treated cells, which served as the control to determine changes to cell viability. Fig 1 shows that naringenin decreased total cell viability when compared to the controls. Our studies show that chronic exposure to naringenin resulted in a time-dependent decrease in cell density with a significant decrease of ~ 30% at 48 h and ~ 50% at 96 h post-treatment when compared to the DMSO controls (Fig 1) and agree with previous studies [21,35].

**Fig 2:**
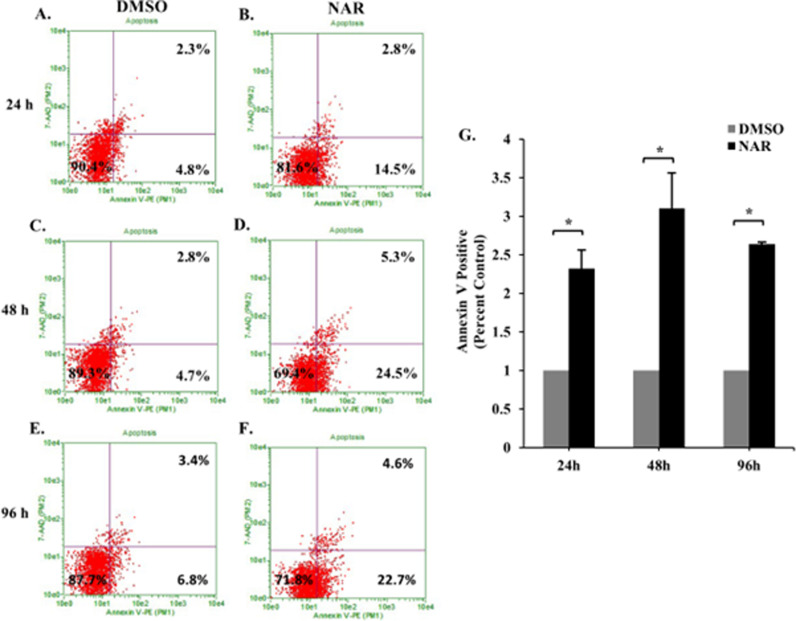
Naringenin induces apoptosis of Tam-R cells. Tam-R MCF-7 cells were treated for 24, 48, or 96 h with DMSO or naringenin. Cells were pelleted, incubated with Guava Nexin reagent and then analyzed using Nexin Software on Guave EasyCyte Flow Cytometer. The bottom left quadrant represents viable cells (Annexin V (-), 7-AAD (-)), the bottom right quadrant represents early apoptotic cells (Annexin V (+) 7-AAD (-)), and the top right quadrant represents late apoptotic cells (Annexin V (+) 7-AAD (+)). Cells were assayed after 24 h (A) DMSO (B) Naringenin, 48 h (C) DMSO (D) Naringenin and 96 h (E) DMSO (F) Naringenin. The data is representative of three independent experiments. G) Total annexin V positive cell count for cells treated with DMSO (gray bar) or naringenin (200 µ M) (black bar) was normalized to untreated control cells for each time point and represented as a fold increase. The data are represented as the mean ± SEM of three independent trials. * p-value ≤  0.05. (S1 Fig. 2A-F).

Naringenin has been shown to promote apoptosis in various cancer cell types [17,35–37]. The current study aimed to provide supportive evidence of naringenin-induced apoptosis in Tam-R MCF-7 cells and to eliminate the possibility of necrotic death. Chronic naringenin treatment in Tam-R cells resulted in approximately a 2-3 fold increase in apoptotic cells (Annexin V positive cells) at 24 h (2.32 ±  0.24), 48 h (3.01 ±  0.46), and 96 h (2.64 ±  0.02) when compared to the DMSO controls (Fig 2). There was little to no change in 7-AAD positive cells among treatments which would indicate little to no necrotic cell death. Together, these findings show that the naringenin induced a decrease in cell density due to increased apoptosis.

### Naringenin impairs cell cycle progression

**Fig 3:**
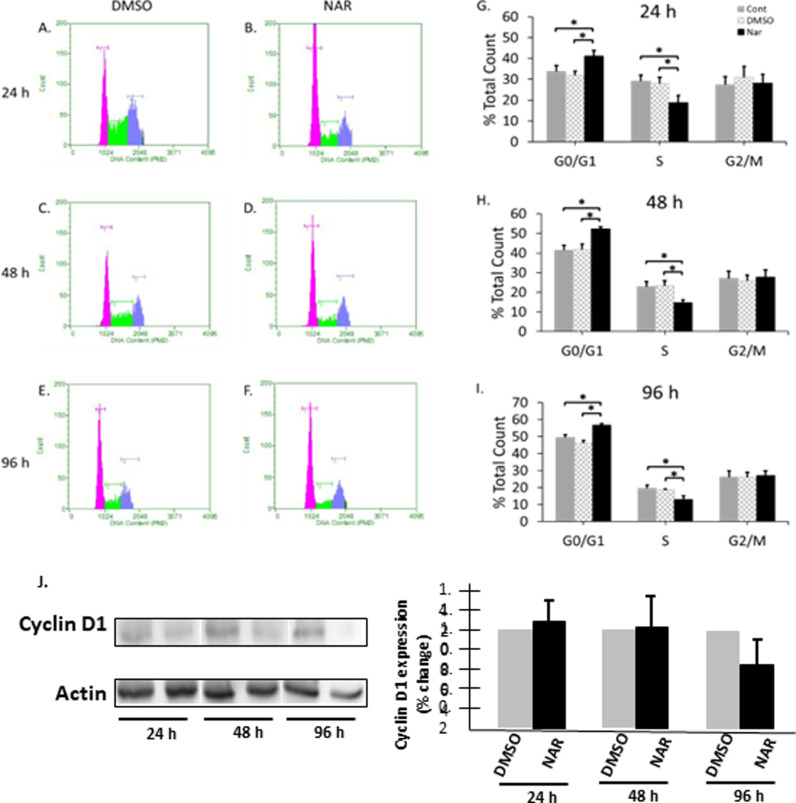
Naringenin impairs cell cycle progression. Tam-R MCF-7 cells were either left untreated (control), or treated with DMSO, or with naringenin (200 µ M) for 24, 48, or 96 h. Cells were collected and then incubated with Guava cell cycle reagent. Cells were analyzed for cell cycle status using CellCycle Software on a Guave EasyCyte Flow Cytometer. Representative cell cycle data for a 24 h treatment A) DMSO B) Naringenin a 48 h treatment C) DMSO, D) Naringenin and a 96 h treatment E) DMSO and F) Naringenin are presented. G-I) Cell count within each cell cycle gated region as indicated in histograms is presented as a percent of total count. The data presented as the mean ± SEM of 4 independent trials. * p-value ≤  0.05. (S2 Fig. 3A-F) J) Tam-R cells were either left untreated (control), treated with DMSO, or with naringenin (200 µ M) for 24, 48, or 96 h and whole cell lysates was assayed for the levels of cyclin D1 and actin by Western blot analysis. Protein levels were quantitated, presented as cyclin D1/actin and normalized to control levels. The data are presented as the mean ± SEM of 3 independent trials S1 File. Raw Images.

Naringenin has also been shown to decrease breast cancer cell proliferation [15,16,21,38]. However, understanding how naringenin elicits these responses is pertinent to the use of naringenin as a potential therapeutic in resistant and non-resistant breast cancer cells. Cell cycle disruption and arrest are linked to the induction of apoptosis [39]. Thus, to determine if naringenin causes cell cycle arrest, Tam-R cells were exposed to naringenin for 24, 48, and 96 h, and cell cycle progression was analyzed using flow cytometry. Starting as early as 24 h post-treatment, naringenin significantly increased the number of cells in G0/G1 by ~ 10% (Control: 33.745 ±  2.92, DMSO: 32.06 ±  1.87, Nar: 41.11 ±  2.83) when compared to controls. Similarly, naringenin decreased cells in S phase by 10% when compared to cells treated with the vehicle alone (Control: 29.155 ±  2.93, DMSO: 27.93 ±  3.15, Nar: 18.69 ±  3.56) (Fig 3A, 3B, 3G). This same pattern persisted at 48 h with an increase in cells in G0/G1 from 41.315 ±  2.63 (Control) and 41.32 ±  2.99 (DMSO) to 52.34 ± 1.06 (Nar) and a decrease in S phase from 22.91 ±  2.62 (Control) and 23.31 ±  2.52 (DMSO) to 14.71 ±  1.54 (Nar) (Fig 3C, 3D, 3H). This pattern continued at 96 h with an increase in cells in G0/G1 from 49.33 ±  1.75 (Control) and 46.14 ±  1.64 (DMSO) to 56.58 ±  1.14 (Nar) and a decrease in S phase from 19.44 ±  1.93 (Control) and 18.65 ±  0.51 (DMSO) to 13.08 ±  1.95 (Nar) (Fig 3E, 3F, 3I). While naringenin did not halt cell cycle progression, it did slow cell cycle progression, as seen by the accumulation of cells in G0/G1 phase. Previous studies have shown that a reduction in the expression of cyclin D can result in G1 cell cycle arrest [40–42]. To determine whether the decrease in cell cycle progression was due to lower levels of cyclin D1, we assayed naringenin treated cells for cyclin D1. We found that naringenin decreased the levels of cyclin D1 by approximately 40% after 96 h of treatment (Fig 3J), indicating a possible mechanism for G0/G1 cell cycle arrest. These findings support the decreased cell growth but does not account for the induced apoptosis.

### Naringenin alters ROS generation

**Fig 4:**
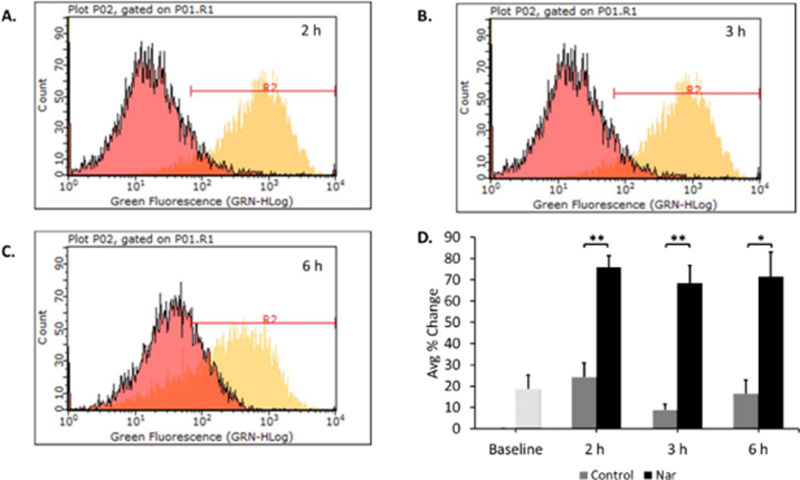
Acute exposure to naringenin increased ROS levels in Tam-R MCF-7 breast cancer cells. Tam-R MCF-7 cells were trypsinized and pelleted. Cell pellets were incubated with CM-H2DCFDA dye for 30 min followed by treatment with DMSO or naringenin (200 μM) and incubated for 2, 3, or 6 h. ROS (total H_2_O_2_) was measured using InCyte software on a GuavaCyte Flow Cytometer. Representative flow cytometry histograms for A) 2 h, B) 3 h and C) 6 h are presented. D) The average percent change in H_2_O_2_ ROS production (represented by dye peaks in the histogram) between control and naringenin were calculated. A baseline measurement of cells with CM-H2DCFDA dye alone was used to normalize the data. The data are presented as the mean ± SEM of 3 independent trials. * p-value ≤  0.05 **p-value ≤  0.005. (S3 Fig. 4A-C).

Our results demonstrate that naringenin slows cell proliferation and induces apoptosis. However, these findings also suggest that naringenin does not induce apoptosis through cell cycle arrest. Therefore, we wanted to determine the underlying mechanism of naringenin-induced apoptosis. Increased ROS has been well-documented to be associated with the induction of apoptosis [43]. Therefore, the total ROS generation in the form of hydrogen peroxide (H2O2) was measured to determine if naringenin increased ROS within the cell. Acute treatment (2,3, and 6 h) with naringenin significantly increased total ROS in Tam-R MCF-7 breast cancer cells from 24.13 ±  6.59 (DMSO) to 76.05 ±  5.21 (Nar) at 2 h, from 8.74 ±  2.73 (DMSO) to 68.28 ±  8.44 (Nar) at 3 h, and from 16.40 ±  6.50 (DMSO) to 71.66 ±  11.50 (Nar) at 6 h (Fig 4D). The peaks represented by naringenin treated cells (shown by the yellow peaks in Fig 4A–4C) significantly shift to the right when compared to the DMSO control cells (shown by the pink peaks in Fig 4A–4C) resulted in an approximately 50-60% increase in H2O2 accumulation across all time points. The data were normalized to baseline with a sample that contained untreated cells with dye alone.

### Naringenin induces production of mitochondrial superoxide

**Fig 5:**
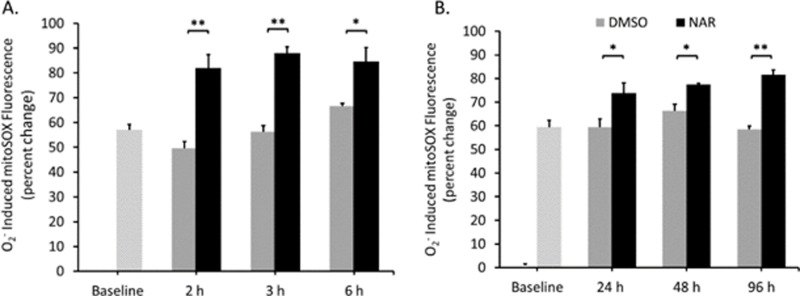
Naringenin increases mitochondrial superoxide in Tam-R MCF-7 cells. Tam-R MCF-7 cells were trypsinized and pelleted. A) Acute treatments: cell pellets were incubated with 5 µ M mitoSOX dye followed by treatment with DMSO or naringenin (200 μM) and incubated for 2, 3, or 6 h. B) Chronic treatments: cells were treated for 24, 48, or 96 h with DMSO or naringenin (200 µ M). Cell pellets were incubated with 5 µ M mitoSOX dye. Superoxide (O_2_^-^) was measured using InCyte software on a GuavaCyte Flow Cytometer. The average percent change in O_2_^-^ accumulation between control cells and naringenin treated cells was calculated. A baseline measurement of cells with mitoSOX dye alone was used to normalize the data. The data are presented as the mean ± SEM of 4 independent trials. * p-value ≤  0.05 **p-value ≤  0.005.

While naringenin increased total ROS, the source of this ROS was still unknown. Low levels of ROS are required for homeostasis and cell signaling, and any deviation from the required levels can result in oxidative stress and cell death [30,44]. ROS is readily generated from sources such as the electron transport chain (ETC) within the mitochondria, the peroxisome, and the endoplasmic reticulum. The mitochondria are the most significant producer of ROS within the cell, due to the ROS generated via oxidative phosphorylation. Although we have shown that naringenin increases total ROS production measured as H2O2, the source of this ROS is unknown. Determining the source of ROS could reveal information about the mechanism of action of naringenin. To determine if naringenin is increasing mitochondrial ROS, in the form of superoxide (O2-), live Tam-R cells were incubated with mitoSOX. In both acute (Fig 5A) and chronic (Fig 5B) treatments, naringenin significantly increased superoxide levels by approximately 20-40% when compared to levels in untreated cells, with slightly lower percentage differences at 24 and 48 h. The observed increase occurs at 2 h and is maintained across all time points. Naringenin significantly increased superoxide ROS levels from 49.55 ±  2.79 (DMSO) to 81.99 ±  5.38 (Nar) at 2 h, from 56.27 ±  2.42 (DMSO) to 88.04 ±  2.49 (Nar) at 3 h, and from 65.0 ±  1.92 (DMSO) to 84.67 ±  5.57 (Nar) at 6 h post-treatment. Naringenin maintained a significant increase in superoxide ROS in chronic treatments with an observed increase from 59.4 ±  3.6 (DMSO) to 73.73 ±  4.36 (Nar) at 24 h, from 66.23 ±  2.9 (DMSO) to 77.33 ±  0.53 (Nar) at 48 h, and from 58.51 ±  1.34 (DMSO) to 81.52 ±  2.05 (Nar) at 96 h post-treatment. Our findings show that mitochondrial-generated ROS (20-40%) can account for a majority of the naringenin induced ROS (50-60% total increase).

### Naringenin did not increase antioxidant enzyme expression

In healthy cells, antioxidant enzymes can convert ROS to non-reactive metabolites and maintain a free radical balance, thus preventing oxidative stress. However, if detoxification processes are altered, or the balance is disrupted, the generated ROS begins to accumulate and leads to oxidative stress and damage. To determine if the naringenin-induced increase in ROS levels is due to altered antioxidant enzyme expression, qRT-PCR was performed. In the current study, four antioxidant enzymes were selected for analysis: glutathione peroxidase (GPx1), superoxide dismutase 1 (SOD1), superoxide dismutase 2 (SOD2), and catalase.

**Fig 6:**
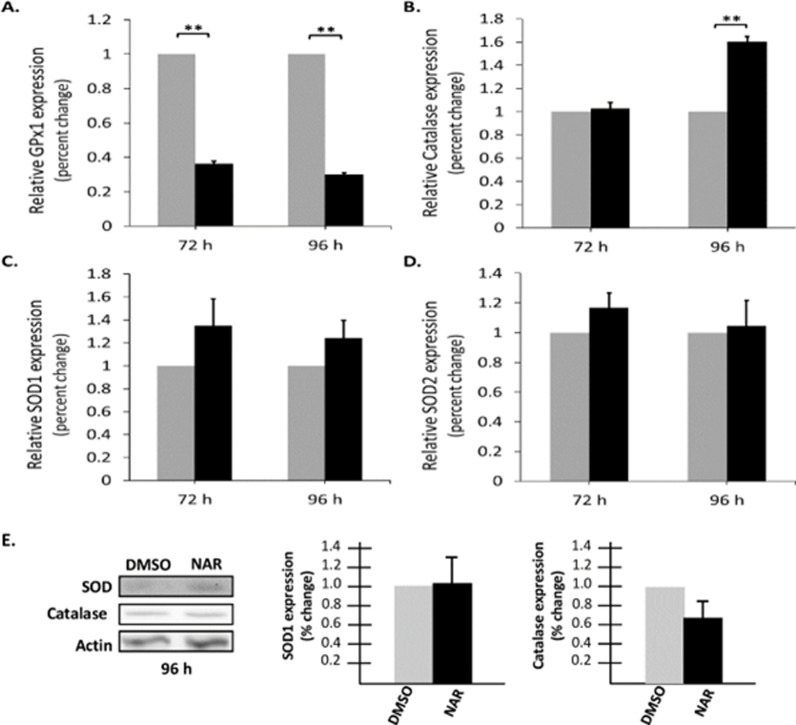
Naringenin did not increase antioxidant enzyme expression. Tam-R MCF-7 cells were treated for 72 or 96 h with DMSO, or naringenin (200 µ M). Total RNA was collected and used for qRT-PCR analysis of comparative gene expression. Expression levels for A) GPx1, B) Catalase, C) SOD1, D) SOD2 were determined and normalized to GAPDH. **p-value ≤  0.005. E) Total protein was isolated from 96 h Tam-R MCF-7 cells treated with either DMSO or naringenin (200 µ M). Western Blot analysis was performed to determine expression of SOD1 and Catalase. Protein levels were normalized to actin levels and data are represented as a percent change relative to DMSO values. The data are presented as the mean ± SEM of three independent trials in triplicates (S1 File. Raw Images).

Total RNA was collected from cells treated with or without naringenin for 72 and 96 h. Relative expression of GPx1 and catalase were significantly changed. GPx1 expression significantly decreased by ~ 50% at 72 h (Nar: 0.36 ±  0.01) and ~ 60% at 96 h (Nar: 0.3 ±  0.01) (Fig 6A) in naringenin-treated cells when compared to DMSO controls. Catalase expression increased by approximately 60% in naringenin-treated cells at 96 h (Nar: 1.6 ±  0.04) compared to the DMSO control (Fig 6B). Both GPx1 and catalase are responsible for detoxing H2O2 [45,46]. While both SOD1 and SOD2 show an increase in mRNA expression, the observed increase was not statistically significant (Fig 6C, 6D). Next, we determined the protein expression of SOD1 and catalase at 96 h (Fig 6E). Similar to SOD1 mRNA expression level, the SOD1 protein levels did not increase significantly. In contrast, the protein levels of catalase did not significantly change even though we observed an increase in the mRNA levels at 96 h. Furthermore, we were unable to detect GPx1 protein at 96 h. These results suggest that the levels of detoxifying enzymes are not increasing with the increasing levels of ROS.

### Naringenin decreases mitochondrial membrane potential

**Fig 7:**
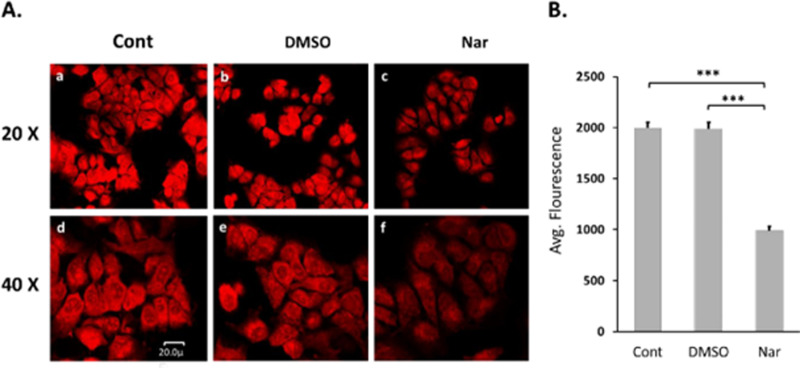
Naringenin decreases mitochondrial function. Tam-R MCF-7 cells were either untreated (control) or treated for 24 h with DMSO or naringenin (200 μM). Cells were stained with MitoTracker red and fixed with paraformaldehyde. A) The same image of MitoTracker red fluorescence was visualized at both 20x and 40x and measured by confocal microscopy. Representative images of three independent experiments are presented (S4 Fig. 7A). B) Average fluorescence intensity across cells was measured and represents mitochondrial membrane potential. The data are an average of at least 3 fields of view from each condition with at least 20 cells per image and expressed as the mean ± SEM of three independent trials in triplicates. ***p-value ≤  0.0005.

Increased ROS is known to cause oxidative stress, membrane damage, and induce apoptosis. Since our results have shown that naringenin increases ROS, this could be the mechanism by which naringenin induces apoptosis. Our findings suggest that the mitochondria are a significant source of increased ROS in naringenin-treated Tam-R cells. Thus, we wanted to determine if there was a change in mitochondrial function that could further explain the mechanism by which naringenin induced apoptosis. It is possible that naringenin induced apoptosis by working through an intrinsic mitochondrial pathway. To address this, we analyzed mitochondrial function. Mitochondrial membrane potential was analyzed using MitoTracker Red in live Tam-R cells that were fixed and visualized using confocal microscopy. When compared to both controls (untreated and DMSO vehicle), naringenin significantly decreased mitochondrial membrane potential (Fig 7A). MitoTracker signal in naringenin-treated cells (1041.36 ±  26.74) is approximately half the signal measured in both control groups (Cont: 1906.4 ±  34.36, DMSO: 1895.88 ±  36.29) (Fig 7B). This decrease in MitoTracker red signal indicates a decrease in mitochondrial function.

## Discussion

Due to the complexity of breast cancer types, treatments, and resistance to commonly used drug therapies, including the widely used drug tamoxifen, there is an increasing demand for alternative therapies. The use of natural, plant-derived compounds as therapeutics and preventatives has gained interest in recent years. Natural treatments that can be consumed easily within the diet or as a supplement would be ideal. Studies have shown the potential use of naringenin as a therapeutic and preventative agent for various cancers [8,10,15,17,47–49]. Many studies have reported pro-apoptotic and anti-proliferative effects of naringenin on cancer cells [10,12,18,20,49,50]. In this study, we showed that naringenin impairs mitochondrial function, resulting in impaired cell growth and induced apoptosis of tamoxifen resistant MCF-7 breast cancer cells. This study further demonstrated that naringenin induces oxidative stress, which, together with diminished mitochondrial function, may explain the observed apoptotic effects of naringenin.

This study aimed to determine the mechanism by which naringenin promotes apoptosis and decreases cell proliferation in Tam-R MCF-7 cells. While the apoptotic and anti-proliferative effects of naringenin have been previously reported [20], the proliferation data was represented only as percent viability; thus, it was essential to analyze the total cell count per treatment to verify changes to proliferation. Our findings show that Tam-R MCF-7 cells treated with naringenin for 96 h had a 50% decrease in total cell count, which verifies previous anti-proliferative studies [14,18,20,35,48]. Further, it was important to verify that naringenin treatment specifically induces apoptosis and not necrosis, which is supported by the data presented here with a 10-15% increase in apoptotic cells at 24, 48, and 96 h. Current literature supports naringenin induction of apoptosis in various cancer cell types [8,16,18,35,51–53], and more specifically a study using MCF-7 cells confirms our findings that a 24 h treatment with 200 µ M naringenin resulted in an approximate 20% increase in apoptotic cells [36]. Our study was able to demonstrate and verify these findings in Tam-R MCF-7 cells, which do not depend on the estrogen receptor (ER) but instead are reported to have alternative growth-promoting pathways such as increased EGFR/HER2 pathway activity, increased kinase pathway activation, and/or altered expression of ER coregulators [54–57]. These findings are in agreement with a recent study showing increased apoptosis in naringenin treated MDA-MB-231 breast cancer cells, which lack ER, PR, and HER2 receptors [58].

To determine cause of the decrease in proliferation and increase in apoptosis, we analyzed cell cycle progression. Alterations in the cell cycle are known to cause cell cycle arrest, potentially leading to programmed cell death [39]. Our study demonstrated that naringenin does not cause cell cycle arrest but does slow cell cycle progression, resulting in a ~ 10% increase in cells in the G0/G1 phase with a corresponding decrease in cells in S phase. Numerous studies have demonstrated that naringenin treatment increases the number of cells in G0/G1 and a corresponding decrease in S phase in various breast cancer cell lines [8,35,59,60]. In contrast, other studies have reported alterations in the G2/M phase of the cell cycle in naringenin treated cells [18,58]. While it is clear that naringenin alters the cell cycle the data point to several different cell cycle phases, which may be due to the different cell lines used. Our findings suggest that naringenin does not alter the cell cycle to induce apoptosis in TAM-R MCF-7 cells. Therefore, we next analyzed a well-known trigger for apoptosis, ROS.

ROS are naturally generated byproducts of cellular respiration via the electron transport chain in the mitochondria. While ROS are required for proper cell function and signaling, a balance must be maintained to prevent high levels of free radicals from accumulating, which can then result in oxidative stress and cell damage [30,45,61]. High levels of ROS result in oxidative stress that leads to membrane damage, DNA mutations, dysfunctional organelles, and decreased metabolic efficiency [31,43,62]. While there are numerous reports of naringenin being an effective antioxidant [26,63,64], along with evidence that flavonoids can inhibit carcinogenesis by suppressing ROS levels, it has been shown that flavonoids can also trigger excessive oxidative stress, which can induce cell death in cancer cells [23]. Naringenin, specifically, has been shown to induce oxidative stress which significantly inhibits the growth of various cancers including breast cancer [14,17,35–37,48,65]. Cancer cells are known to have increased ROS generation and re-programmed metabolism [66,67]. These re-programmed events may alter the response of the cell to certain compounds, such as flavonoids, which could in turn explain why naringenin acts as a pro-oxidant rather than an antioxidant in cancer cells. To investigate if naringenin acts as a pro-oxidant in Tam-R MCF-7 cells, H2O2 levels were determined as a measure of total ROS production. Our results demonstrate that naringenin significantly increased H2O2 (~60%) after just 2, 3, and 6 h. This suggests that the induced ROS from naringenin treatment results in significantly increased levels of ROS, which may lead to cell death.

We next aimed to determine the source of the ROS using a MitoSOX assay to measure mitochondrial-generated superoxide. ROS are key signaling molecules involved in cell death, specifically via the intrinsic mitochondrial pathway [44], and damage from superoxide anions is involved in cancer formation [68,69]. We demonstrated for the first time that naringenin significantly increases superoxide production across acute (2, 3, and 6 h) and chronic (24, 48, and 96 h) treatments in Tam-R MCF-7 breast cancer cells. A previous study reported increased superoxide anion and mitochondrial dysfunction in ER^—^ breast cancer cells but did not have this same effect in ER + cells [37]; however, because Tam-R MCF-7 cells bypass ER-dependent growth, naringenin would likely elicit a response similar to that seen in ER^—^ cells. Together, these findings suggest that naringenin induces oxidative stress through increased mitochondrial superoxide. These findings are significant because they verify naringenin as a pro-oxidant in cancer cells and provide novel evidence that naringenin not only increases ROS generation in Tam-R MCF-7cells but does so via mitochondrial ROS.

Cells naturally combat high levels of ROS through antioxidant enzymes [30]. This checks and balances system is essential to maintain cell function. GPx1 is an essential antioxidant enzyme that is crucial for maintaining cellular redox balance by controlling ROS levels and has a multifaceted role in cancer [70]. The role of GPx1 in various cancers is complex, however, its overexpression significantly impacts tumor behavior, influencing processes like cell proliferation and apoptosis [70]. Its expression levels in tumor tissues shows a negative correlation with overall survival in breast, gastric, glioma, and leukemia cases, whereas in pancreatic cancer, it indicates a favorable prognosis [70]. Our study reports that naringenin treatment significantly decreased the expression of GPx1 mRNA which is essential for detoxifying H2O2. However, the protein levels of SOD1 and catalase were unchanged in the presence of increased ROS levels and may indicate that naringenin is preventing cells from responding. Naringenin induced an increase in ROS generation and a decrease in the ability of the cells to detoxify ROS, which resulted in an increase in ROS accumulation and measurable cell damage. To determine if this increase in mitochondrial superoxide altered mitochondrial function, we measured changes in membrane potential. Membrane potential is essential for proper mitochondrial function. A decreased membrane potential indicates a leaky membrane, which is caused by free radicals, which in turn induces the intrinsic apoptotic pathway [44]. In the presence of naringenin, there was a significant decrease in membrane potential, which indicates a significant decrease in mitochondrial function. Previous studies report cancer cells have elevated intrinsic ROS levels due to changes in metabolic demands, mitochondrial dysfunction, or cell damage [32], which makes this system especially interesting as a potential target for therapeutics.

Although previous studies have suggested a mechanism of action for naringenin in cancer cells, our study demonstrates that naringenin is capable of impairing mitochondrial function in Tam-R MCF-7 cells. It has been shown that naringenin-induced apoptosis in human promyeloleukemia HL-60 cells was linked to decreased mitochondrial membrane potential, resulting in mitochondrial dysfunction and the activation of the intrinsic apoptotic pathway [71]. This decrease in mitochondrial function is likely a result of increased ROS, which in turn damages membrane integrity, resulting in a leaky membrane and ultimately promoting apoptotic signaling. A study using human pancreatic cancer SNU-213 cells showed that naringenin provoked a significant increase in ROS, resulting in apoptotic cell death (~30%), which is similar to the apoptotic cell death observed in our study (Fig 2) [17]. Similarly, a study that evaluated the cytotoxic, genotoxic, and apoptotic effects of naringenin on various cancer cell types (MCF-7, HT-29, PC-12, and L-929 cells) found that naringenin caused a decrease in cell viability, induced apoptotic cell death, and promoted a significant increase in ROS in a dose-dependent manner [36]. This same study reported that 200 µ M naringenin resulted in an approximately 30% increase in apoptotic cells and a 2.5-fold increase in ROS [36], which is consistent with our current study. Another study delved into the potential anti-cancer properties of naringenin by investigating its effects on ROS production and the mechanism underlying cell death induction in NSCLC cells [72]. Upon treatment with naringenin, both A549 and H1299 cells exhibited a notable dose-dependent increase in intracellular ROS levels. Pre-treating the cells with ROS scavengers NAC and catalase significantly mitigated the naringenin-induced cleavage of caspase 9, caspase 3, and PARP, while also demonstrating a reduction of naringenin-mediated cell death [72]. These findings collectively suggest that naringenin facilitates apoptosis in cancer cells through ROS induction [72]. It is evident that naringenin can promote ROS generation, which is the likely path for naringenin-induced apoptosis through mitochondrial signaling and dysfunction. We show for the first time that naringenin is capable of promoting these same effects in Tam-R MCF-7 breast cancer cells, which bypass the traditional growth pathways.

With an increase in ROS, mainly superoxide, which is highly reactive, the electron gradient required for proper mitochondrial function is disrupted. A decrease in glutathione peroxidase 1 significantly decreases the detoxification process and further contributes to free radical accumulation. This buildup of ROS likely causes mitochondrial dysfunction and results in decreased proliferation and the induction of apoptosis. These studies demonstrate that naringenin is a potential therapeutic for tamoxifen resistant breast cancer cells. Since naringenin is capable of further increasing ROS levels in cancer cells to induce apoptosis, it may serve as a favorable drug treatment.

Further studies are needed to better understand the underlying mechanism by which naringenin promotes mitochondrial dysfunction and increased ROS production. If naringenin is altering mitochondrial function, resulting in increased ROS, this would suggest that naringenin would be an ideal treatment for various cancers as well as drug-resistant cancers. This growing body of evidence supports the use of naringenin as a universal cancer therapeutic.

## Supporting information

S1 Fig 2A–F: Three independent trials of cell apoptosis analyzed using Nexin Software on Guave EasyCyte Flow Cytometer for 24, 48, or 96 h in Tan-R cells treated with DMSO or naringenin. (PDF)

S2 Fig 3A-F: Trials of cell cycle analysis using CellCycle Software on a Guave EasyCyte Flow Cytometer for Tam-R cells treated with DMSO or Naringenin for 24, 48 and 96 h. (PDF)

S3 Fig 4A-C: ROS (total H2O2) was measured using InCyte software on a GuavaCyte Flow Cytometer in cells treated with DMSO or naringenin for 2, 3, or 6 h for three independent trials. (PDF)

S4 Fig 7A: MitoTracker red fluorescence images at both 20x and 40x by confocal microscopy for two independent experiments. (PDF)

S1 File Raw Western blot images. (PDF)

S2 File Raw data. (PDF)
