# Supplementary material for: Naringenin impairs mitochondrial function via ROS to induce apoptosis in tamoxifen resistant MCF-7 breast cancer cells
Source: PLoS One. 2025 Apr 3;20(4):e0320020. doi: 10.1371/journal.pone.0320020 (PMC11967926; doi:10.1371/journal.pone.0320020)

S1 Fig. 2A-F

Trial 1 24 h DMSO

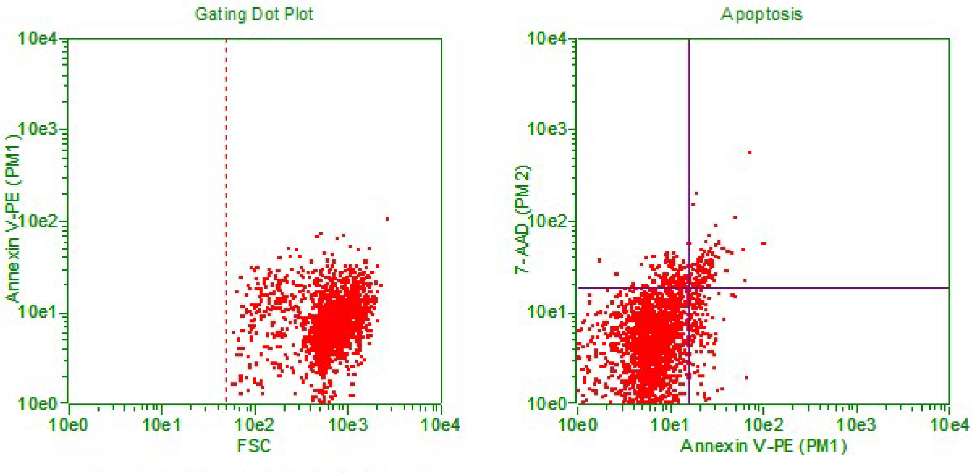

Trial 1 24 h NAR

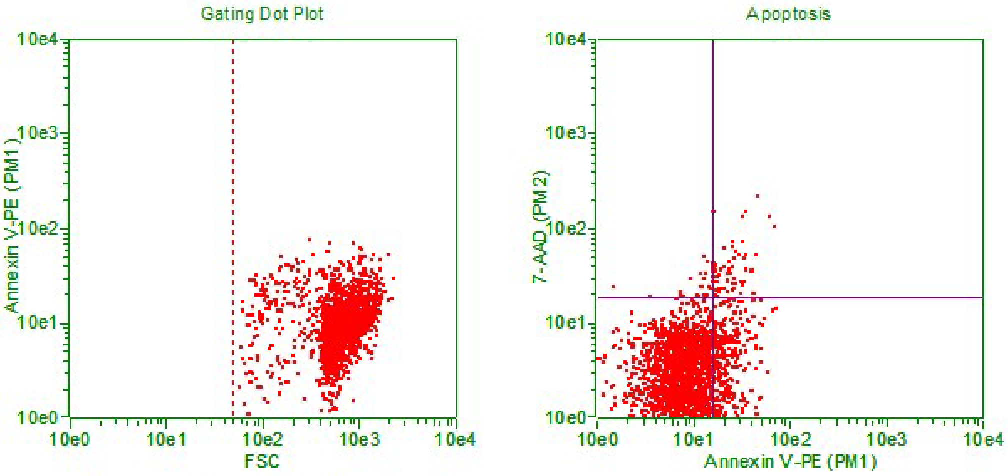

Trial 1 48 h DMSO

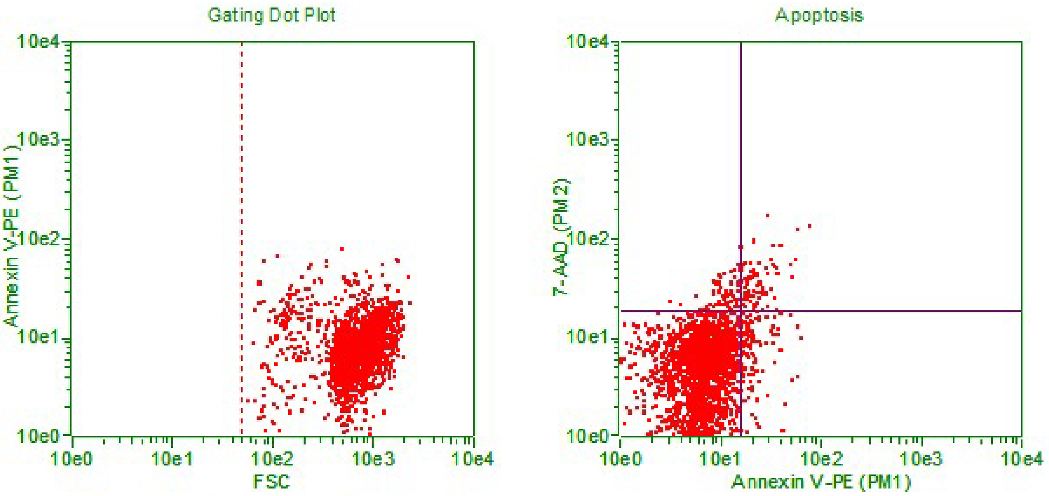

Trial 1 48 h NAR

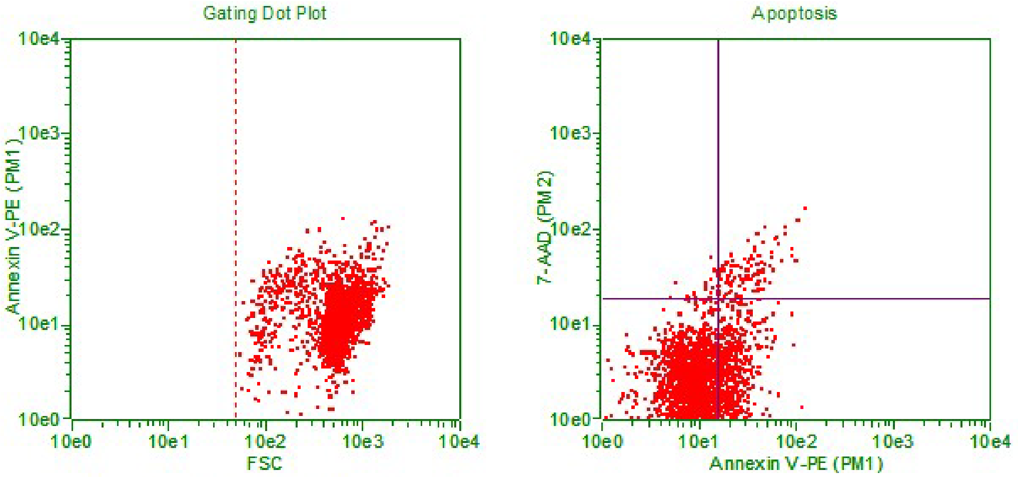

Trial 1 96 h DMSO

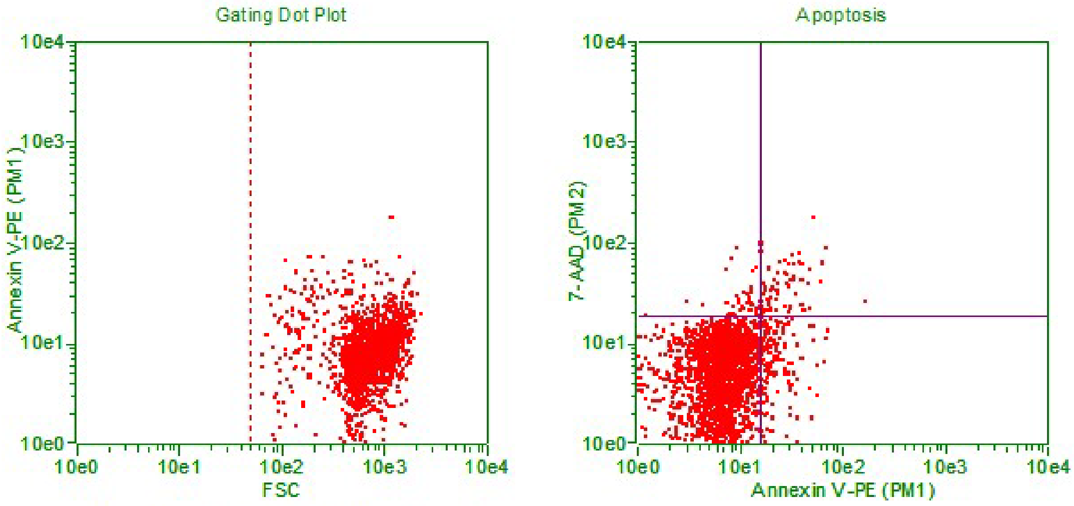

Trial 1 96 h NAR

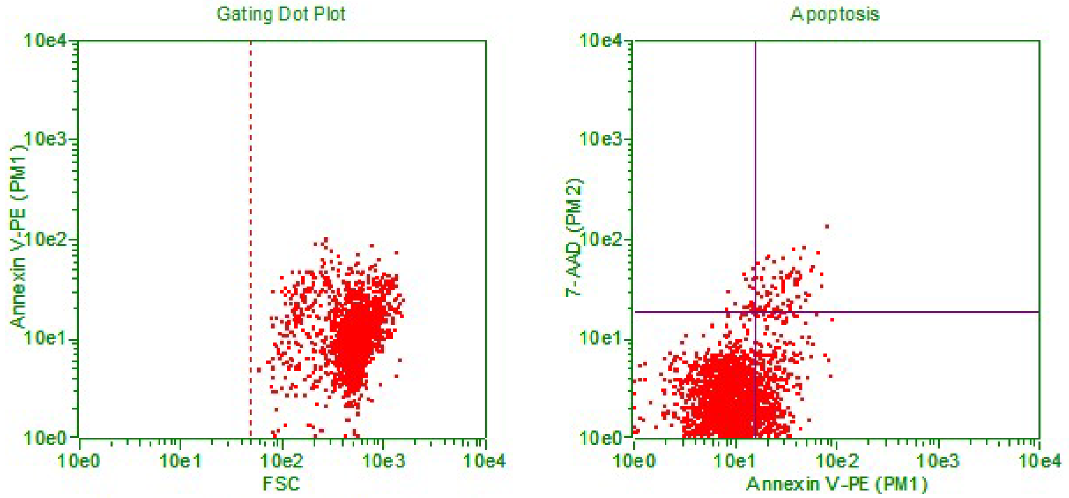

Trial 2 24 h DMSO

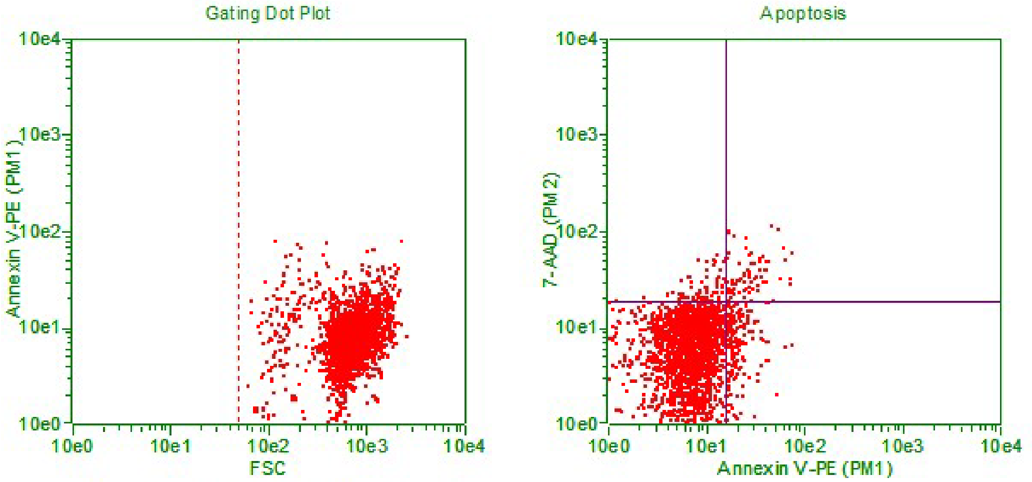

Trial 2 24 h NAR

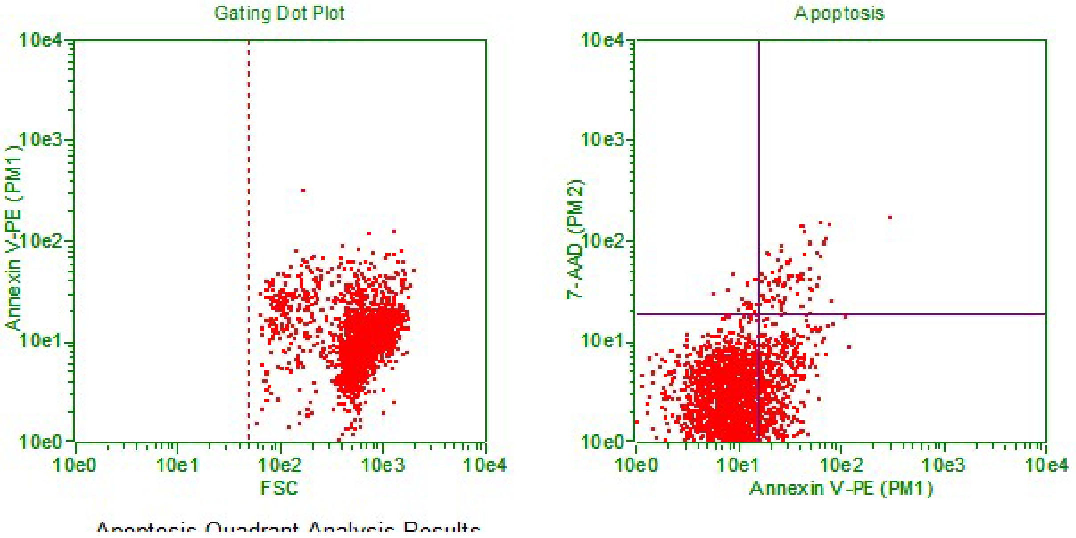

Trial 2 48 h DMSO

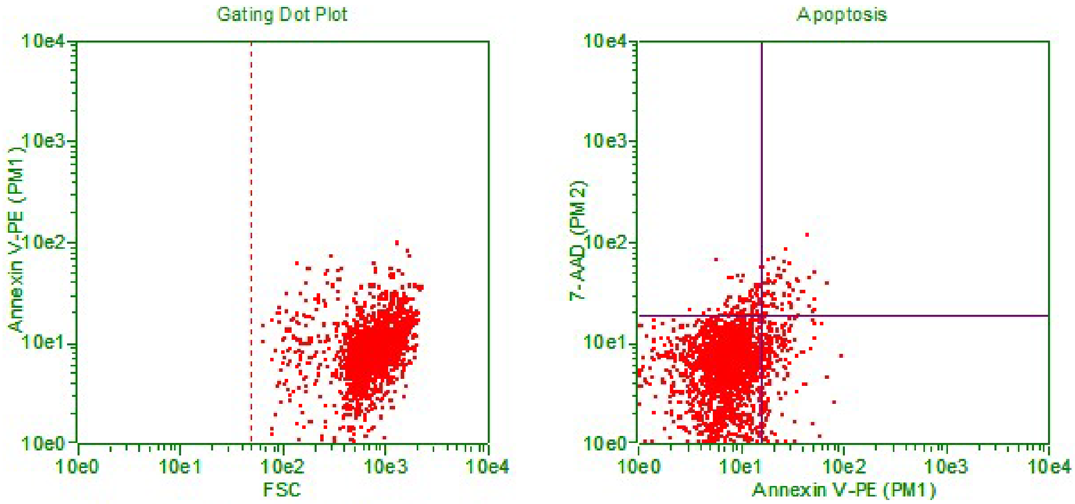

Trial 2 48 h NAR

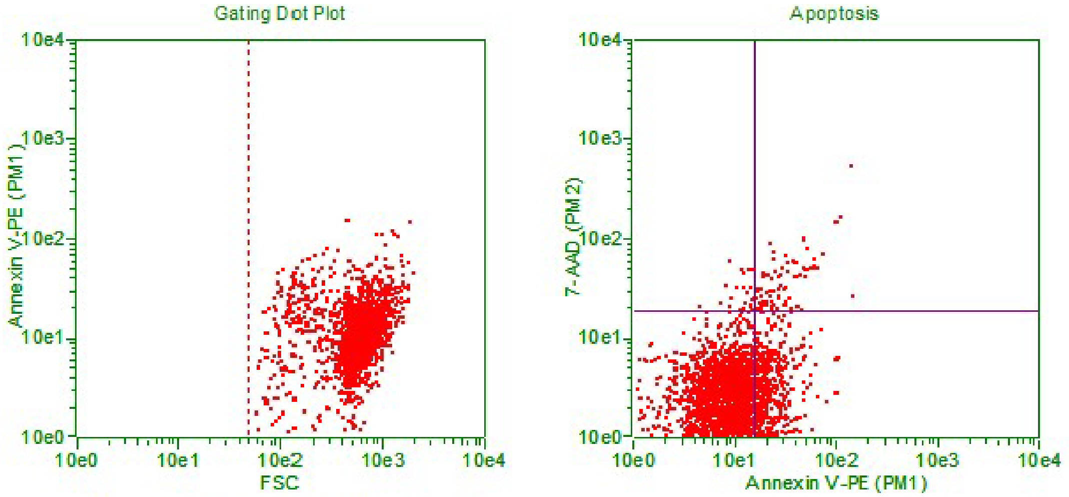

Trial 2 96 h DMSO

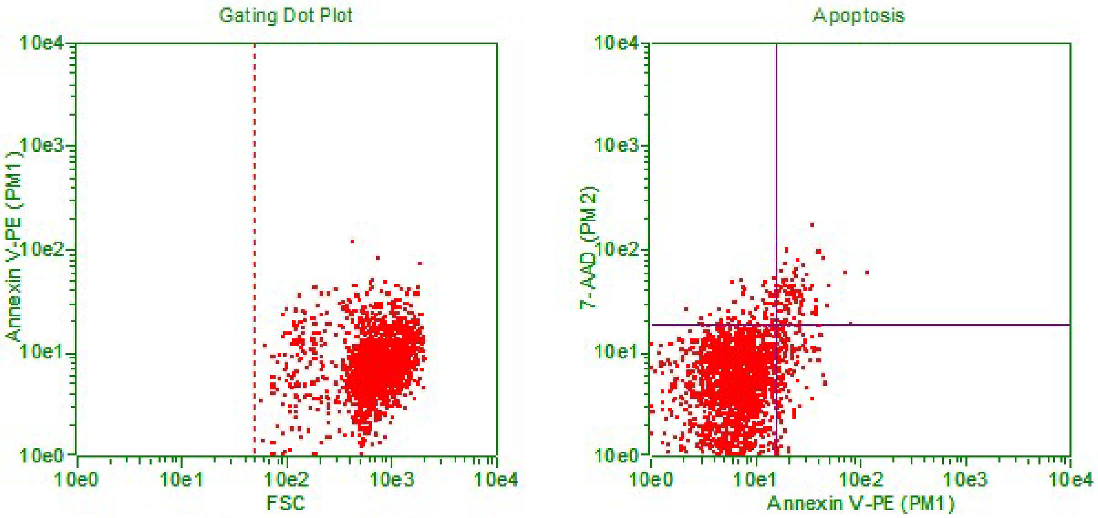

Trial 2 96 h NAR

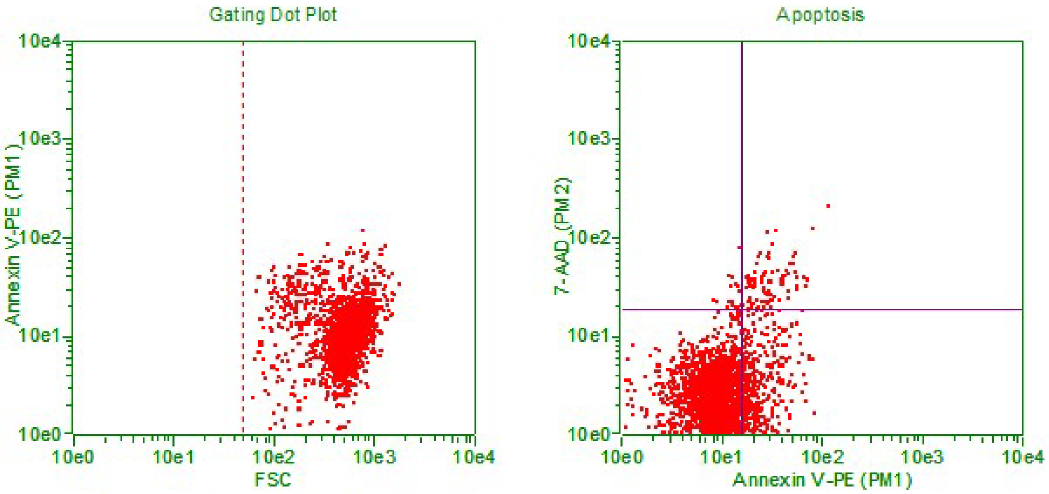

Trial 3 24 h DMSO

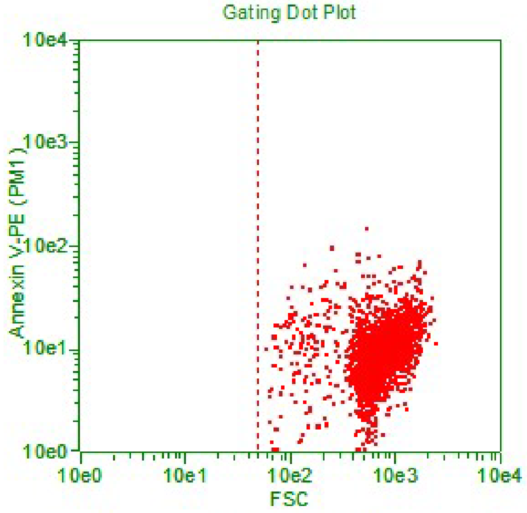

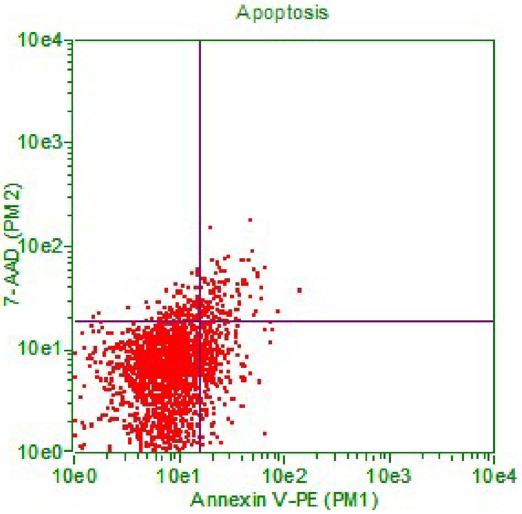

Trial 3 24 h NAR

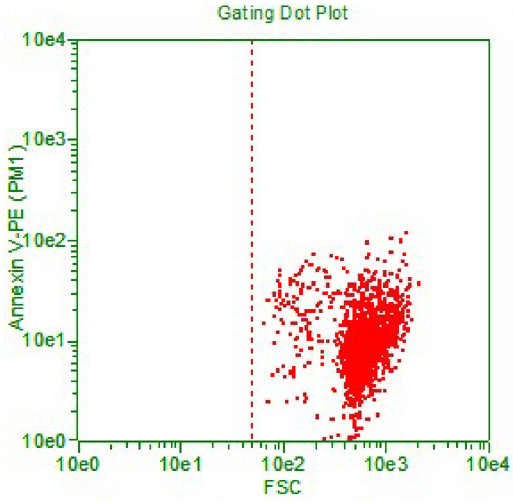

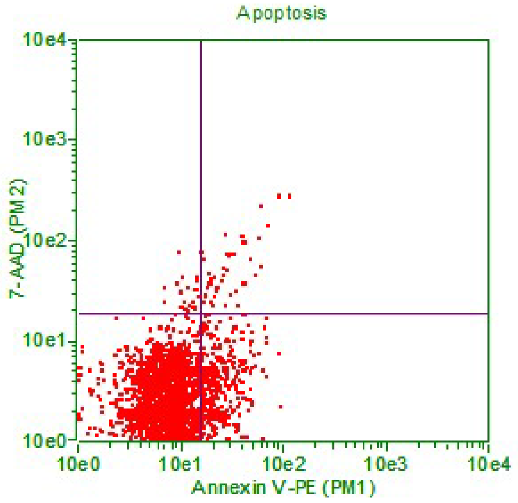

Trial 3 48 h DMSO

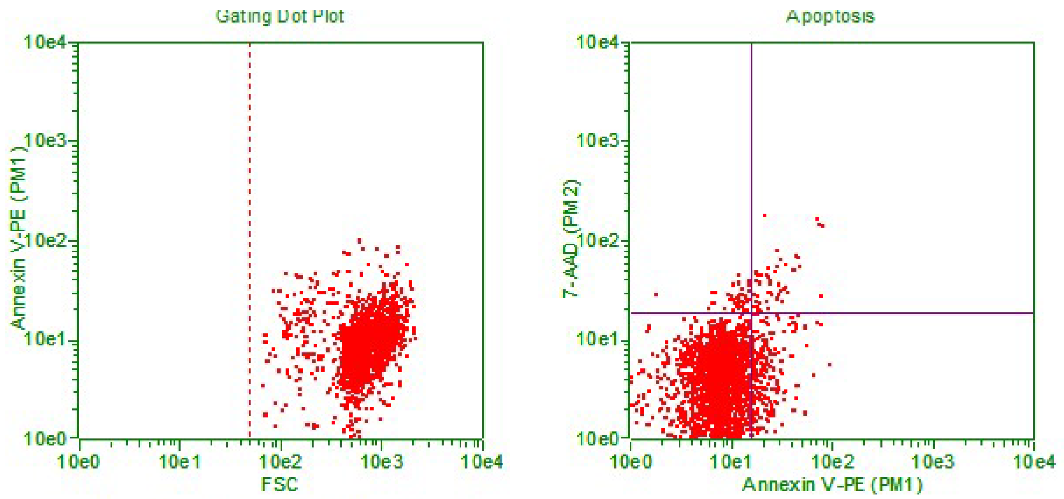

Trial 3 48 h NAR

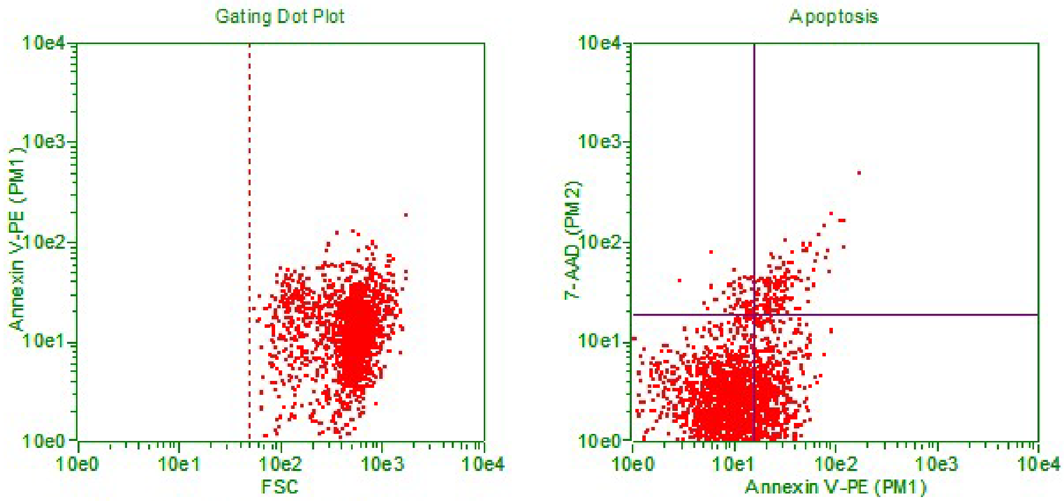

Trial 3 96 h DMSO

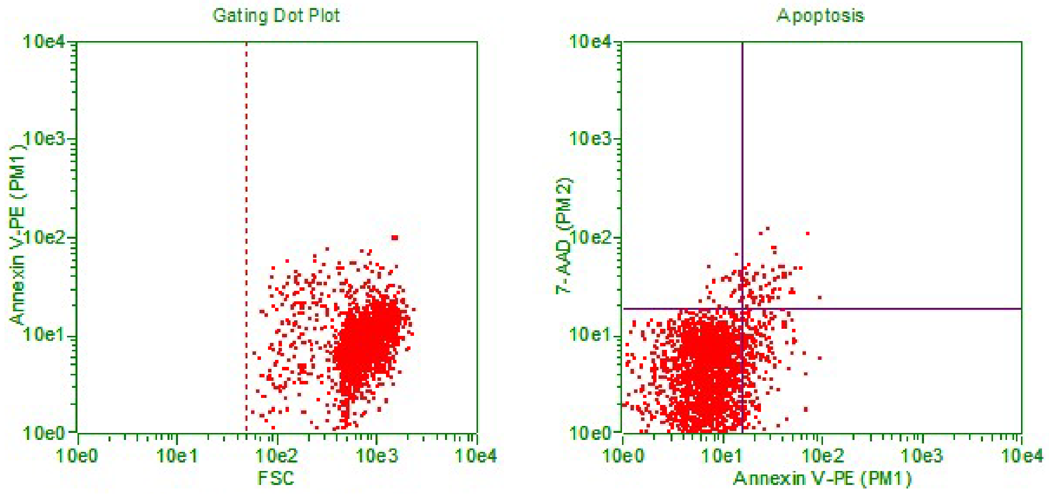

Trial 3 96 h NAR

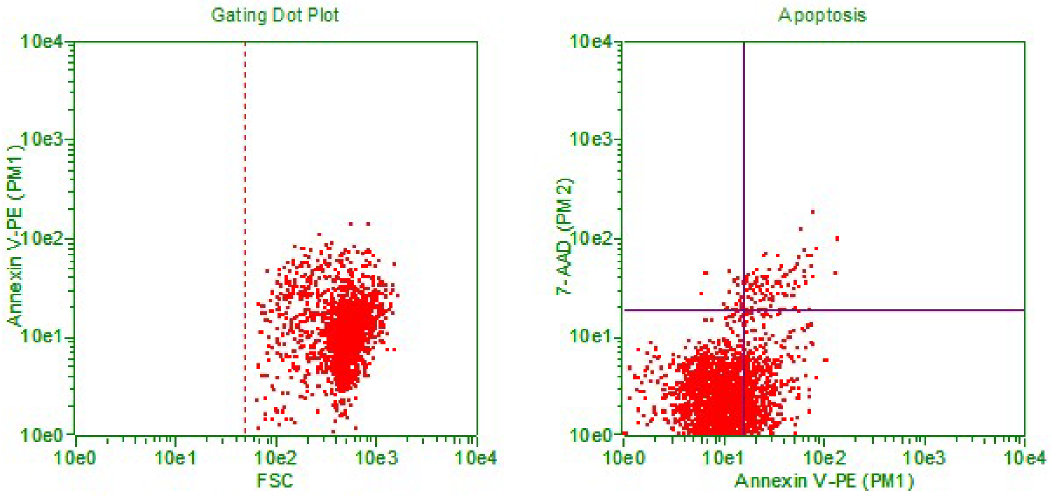

Supplement: S1 Fig — (PDF) [file pone.0320020.s001.pdf]
