# Supplementary figures and images for: Naringenin impairs mitochondrial function via ROS to induce apoptosis in tamoxifen resistant MCF-7 breast cancer cells
Source: PLoS One. 2025 Apr 3;20(4):e0320020. doi: 10.1371/journal.pone.0320020 (PMC11967926; doi:10.1371/journal.pone.0320020)

S3. Fig 4A-C.

Trial 1

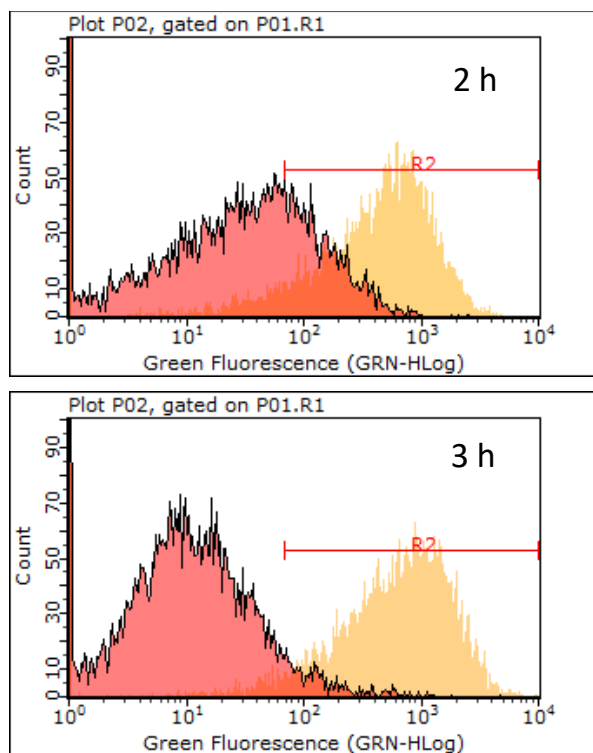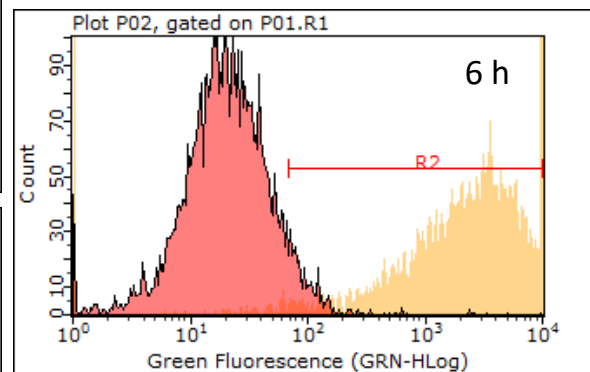

**Trial 2**

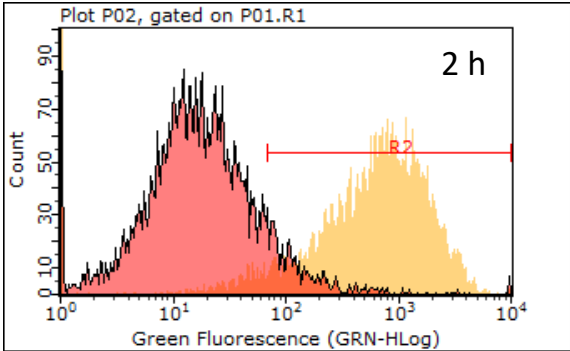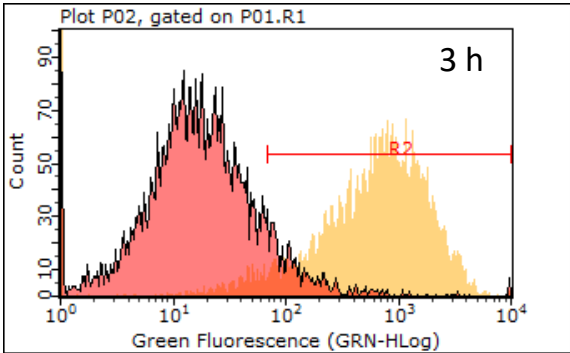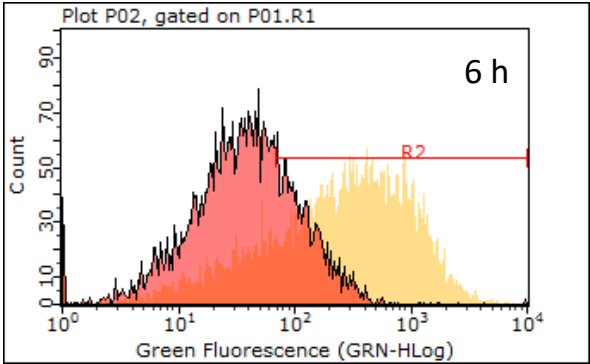

### Trial 3

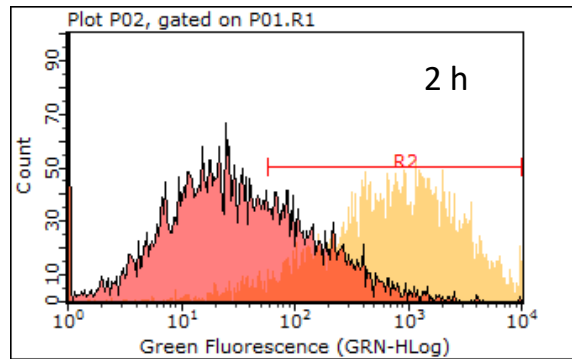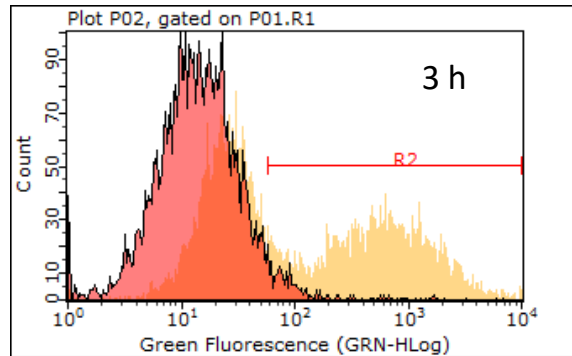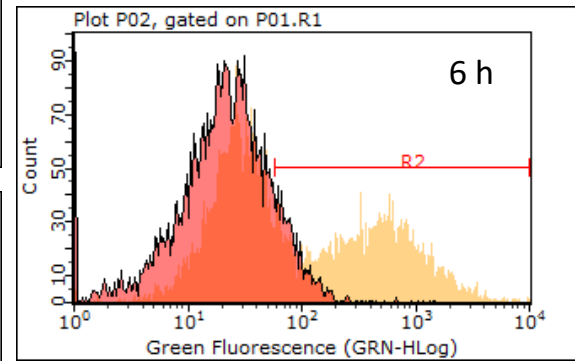

Supplement: S3 Fig — (PDF) [file pone.0320020.s003.pdf]

S1 Fig.3J

Cyclin D1 Actin

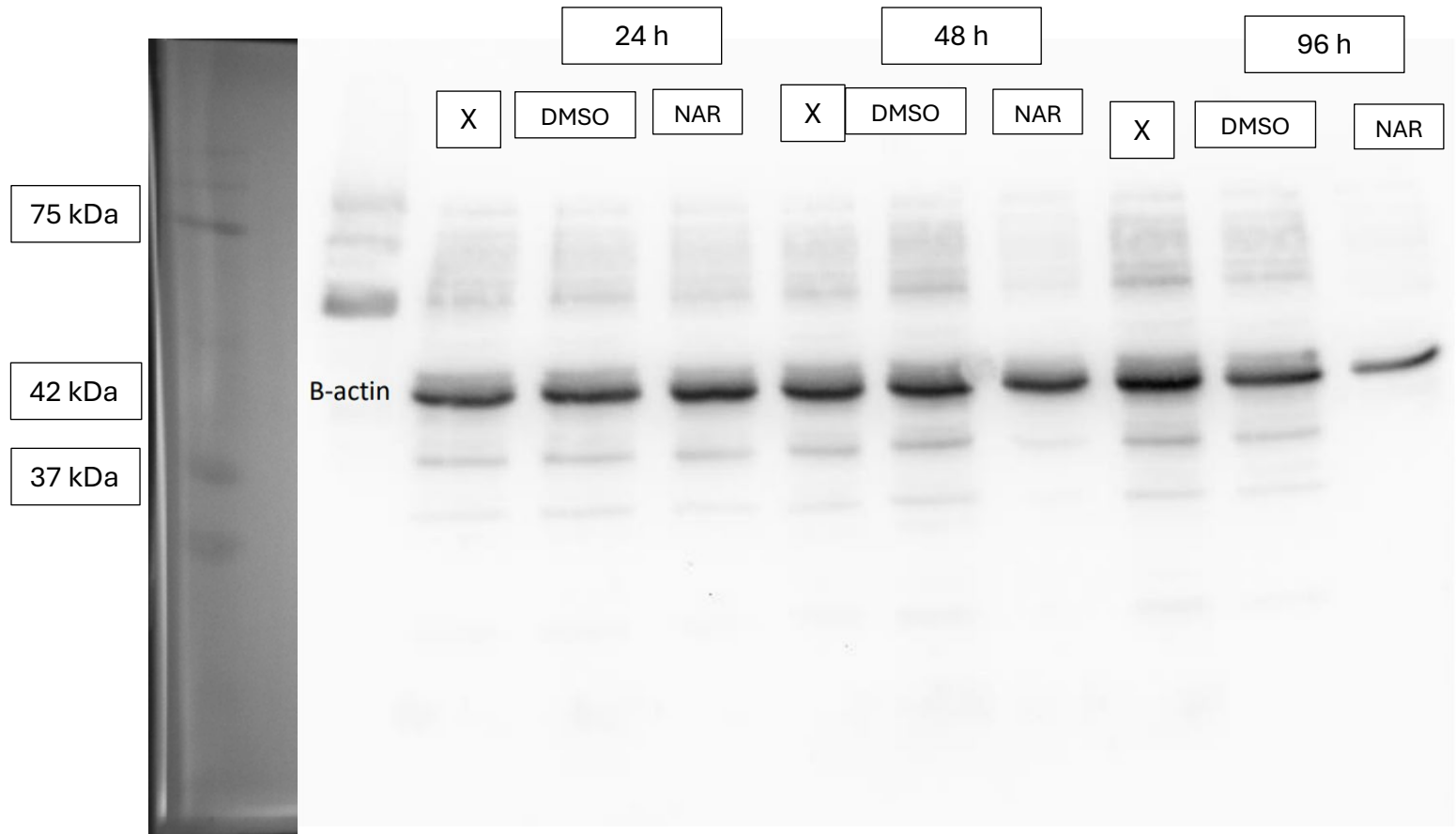

Cyclin D1

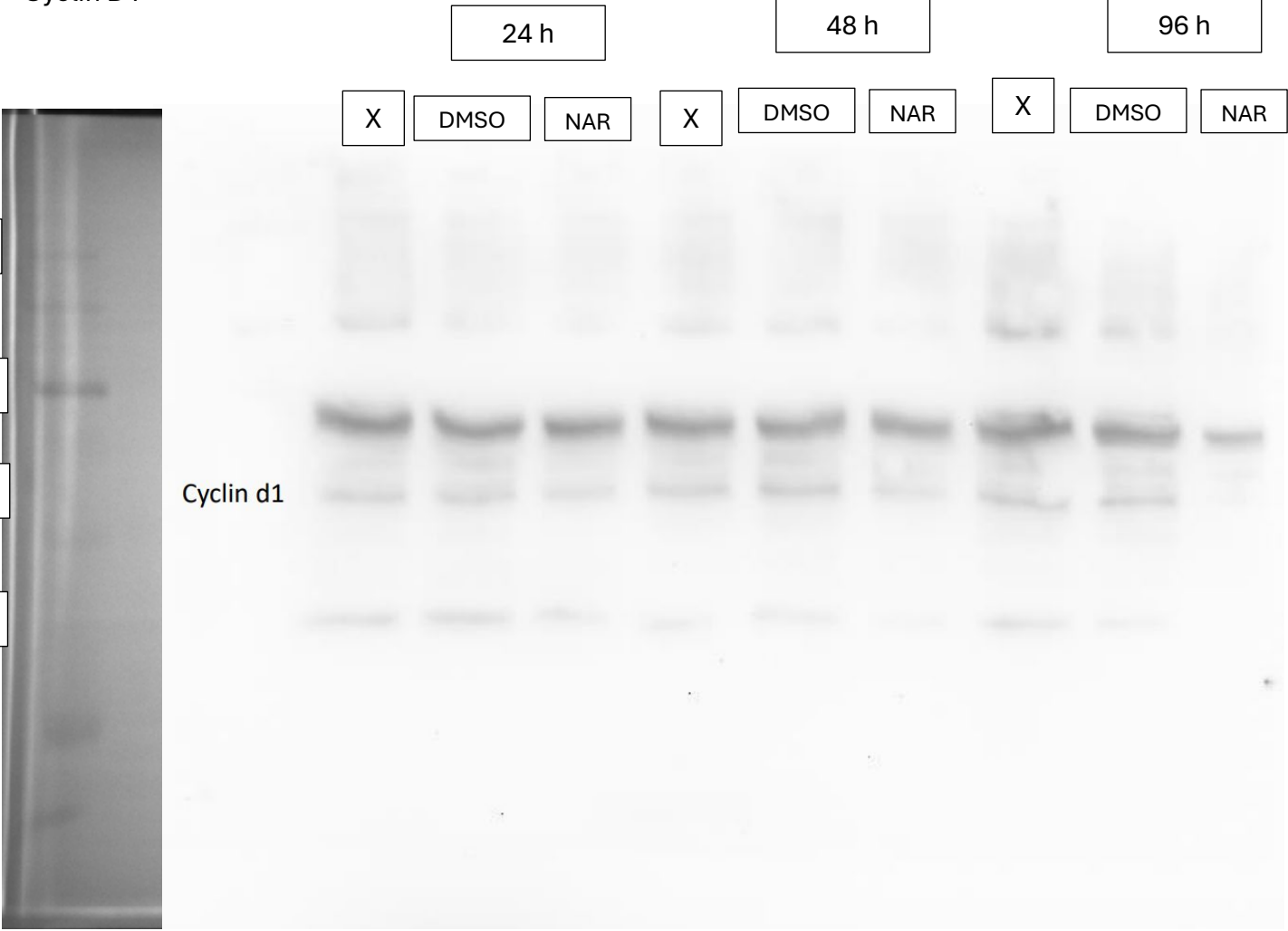

S1 Fig. 6E

B-Actin

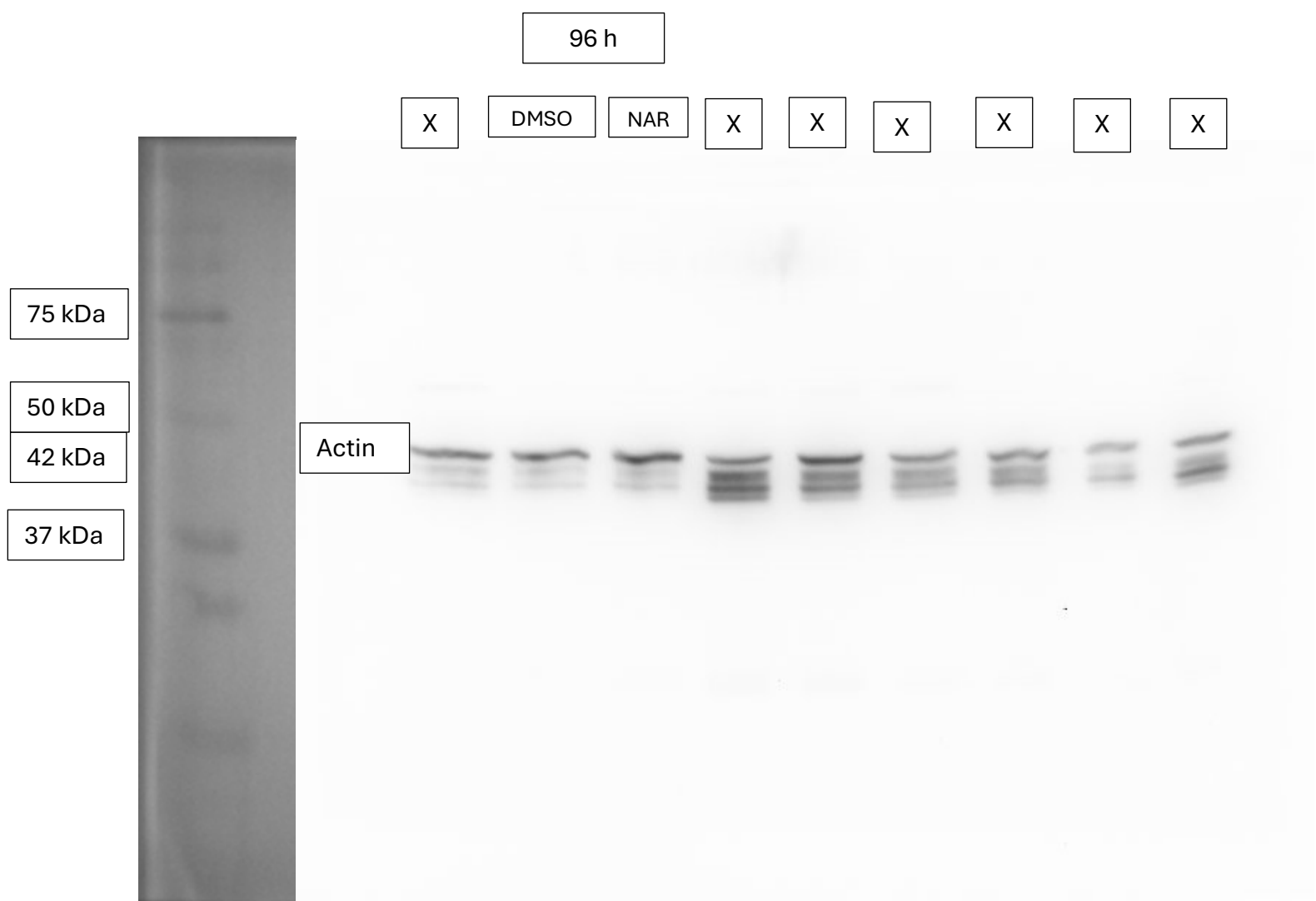

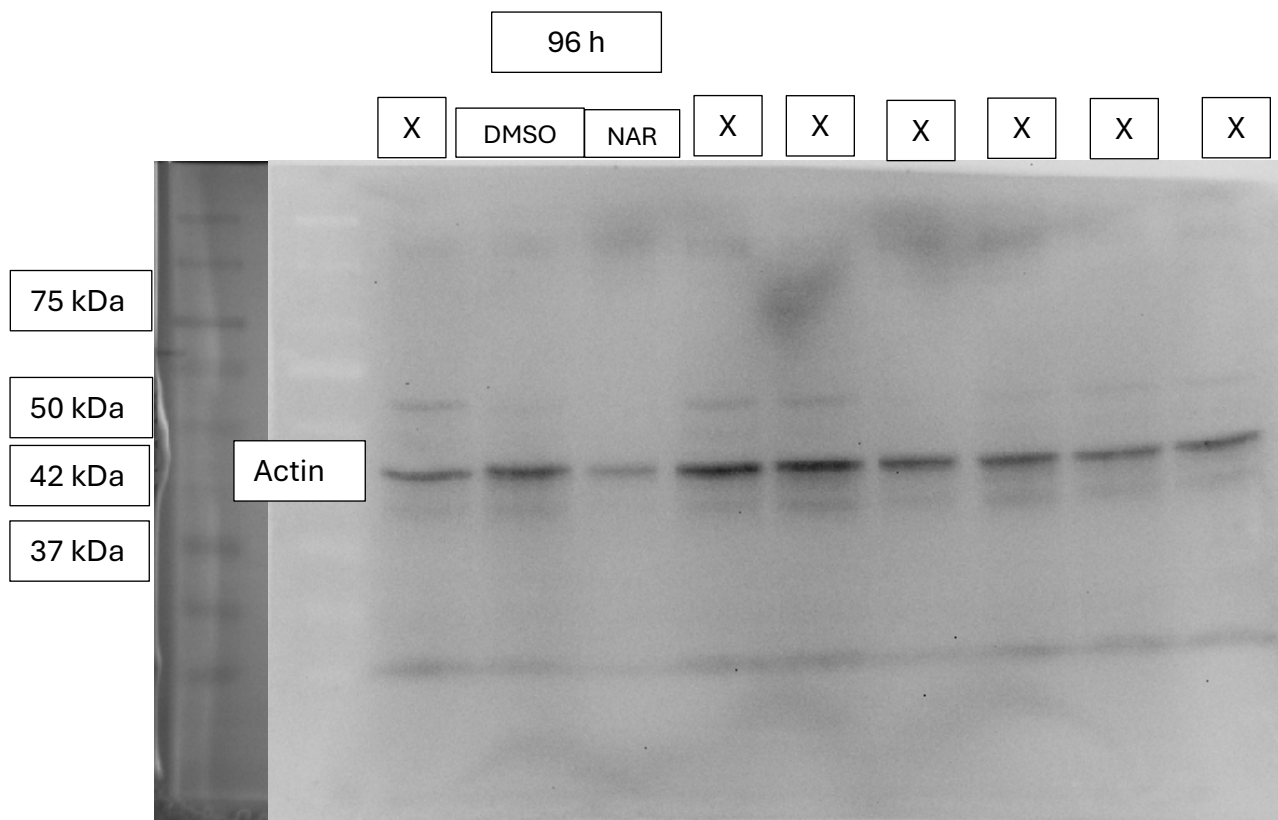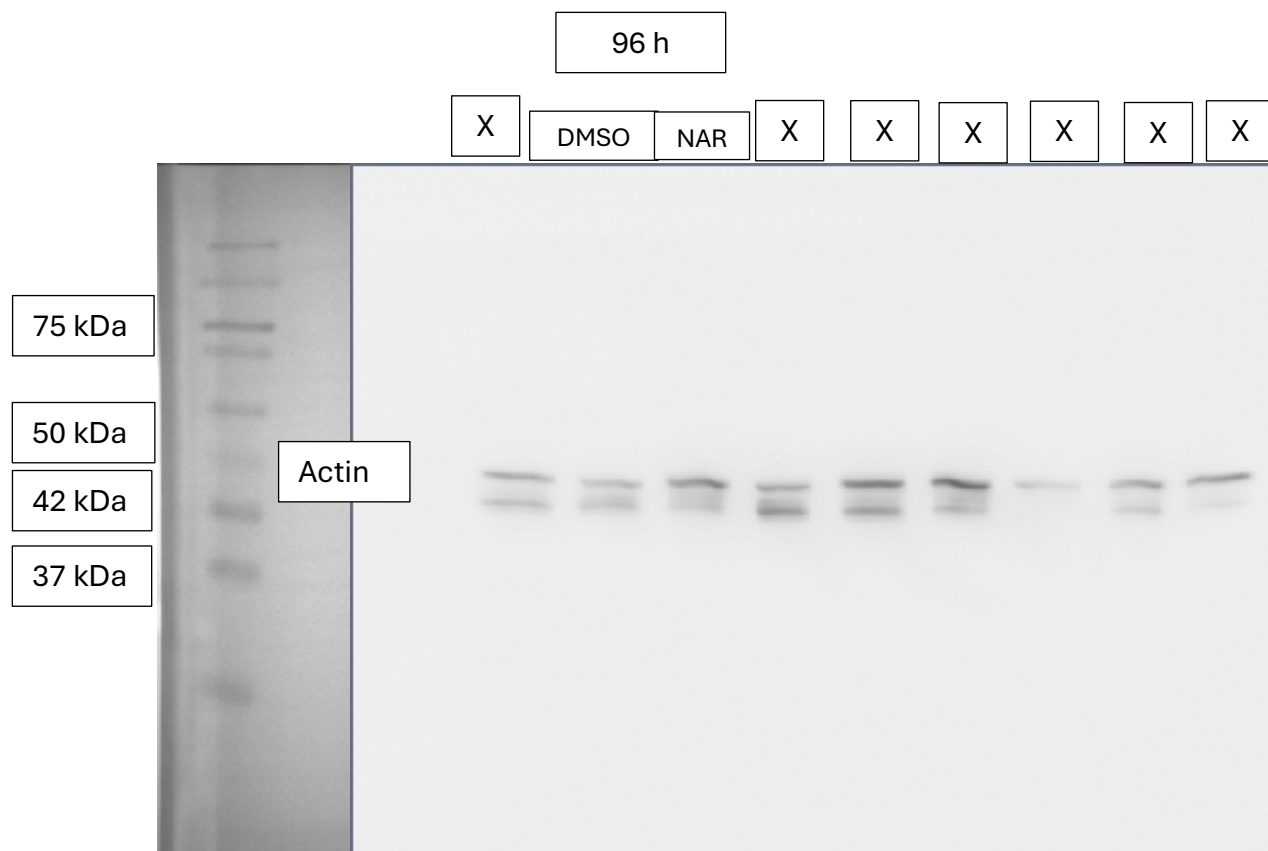

Catalase

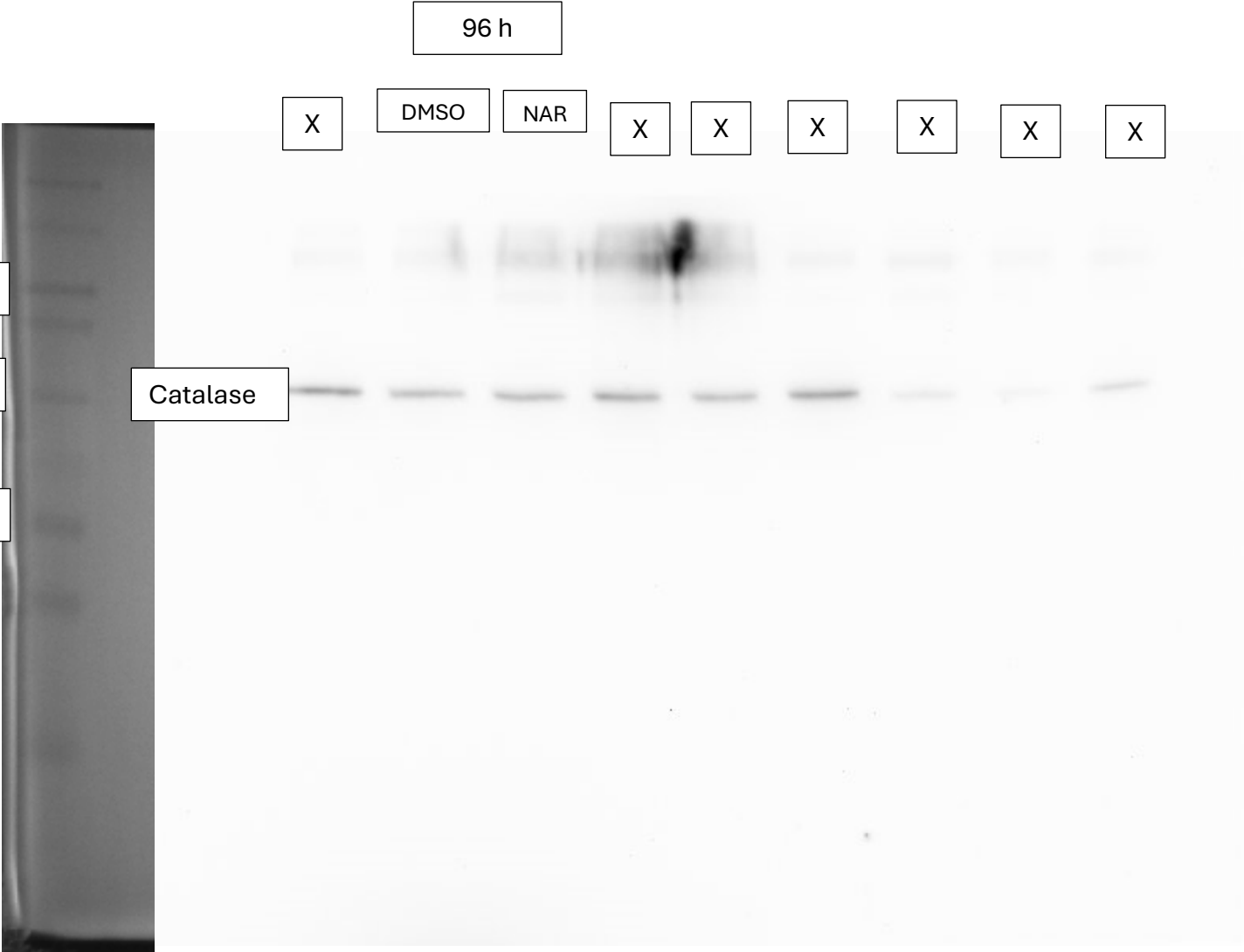

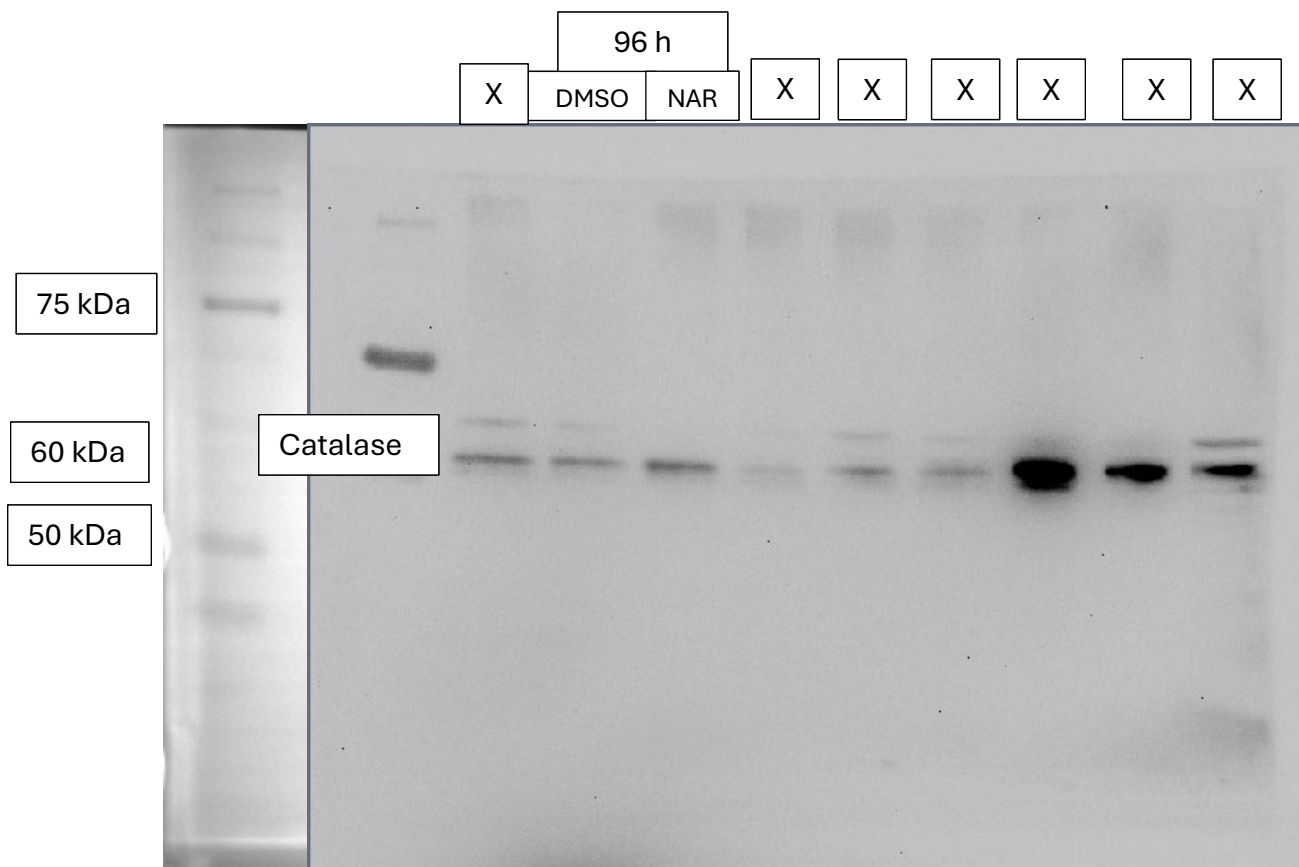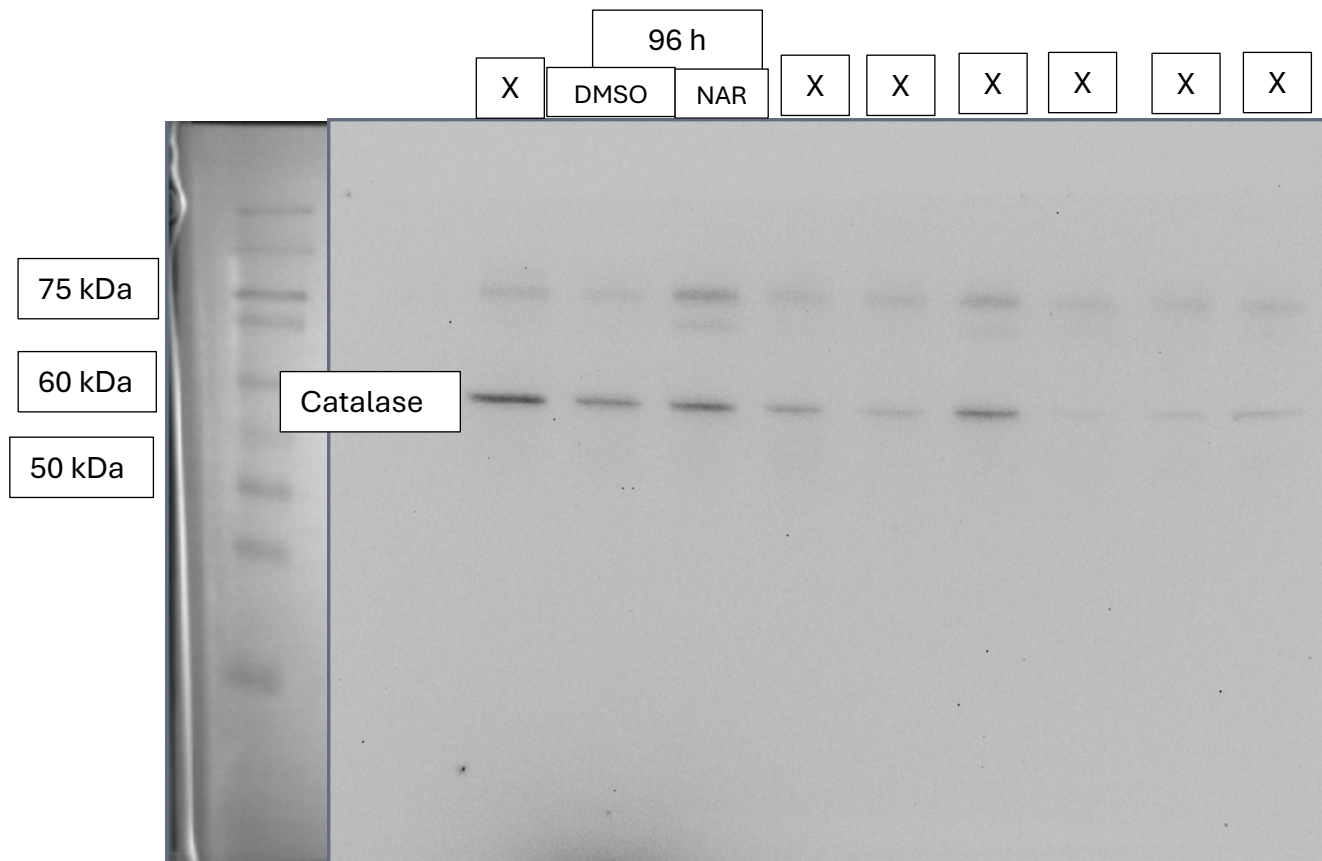

SOD1

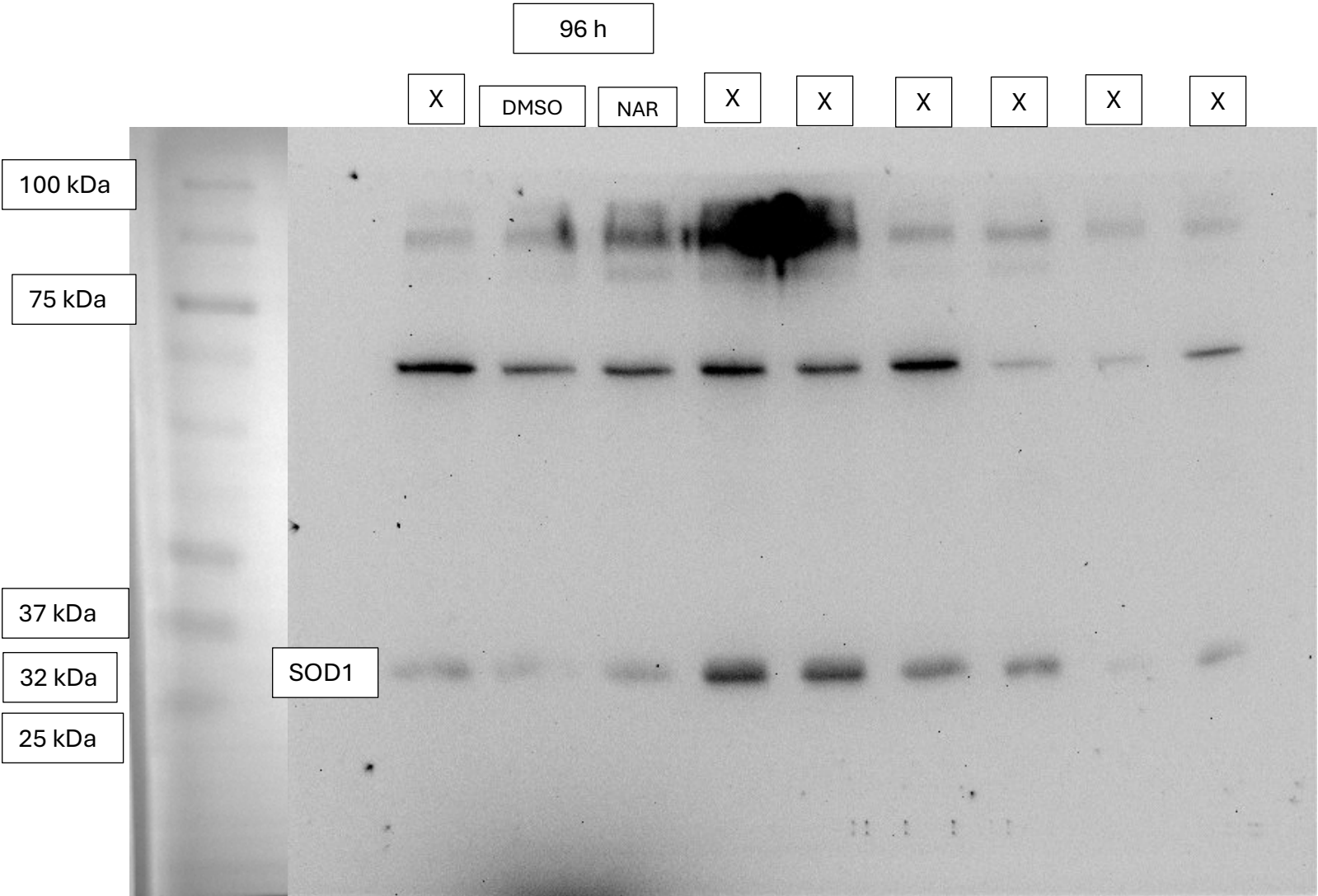

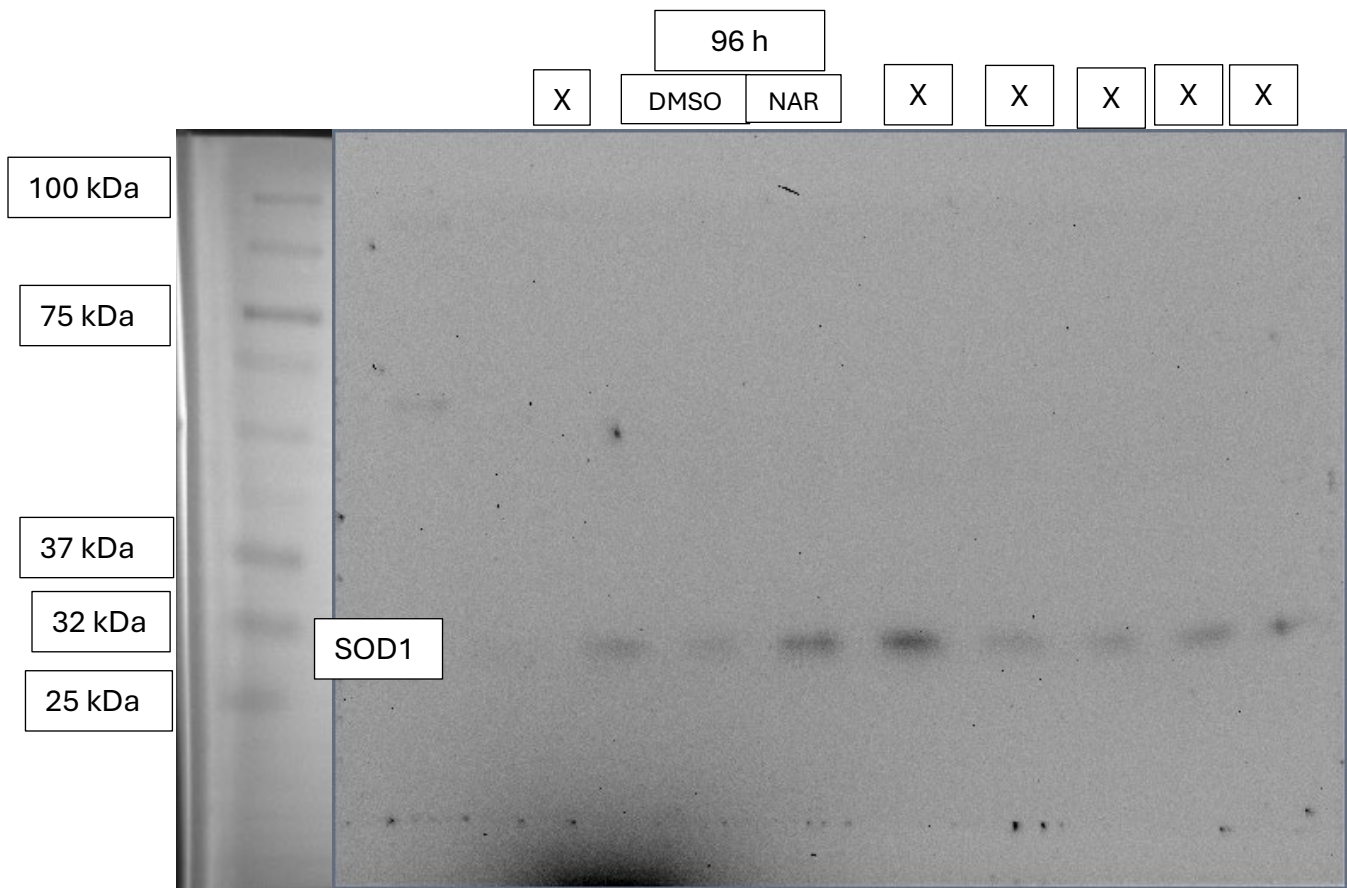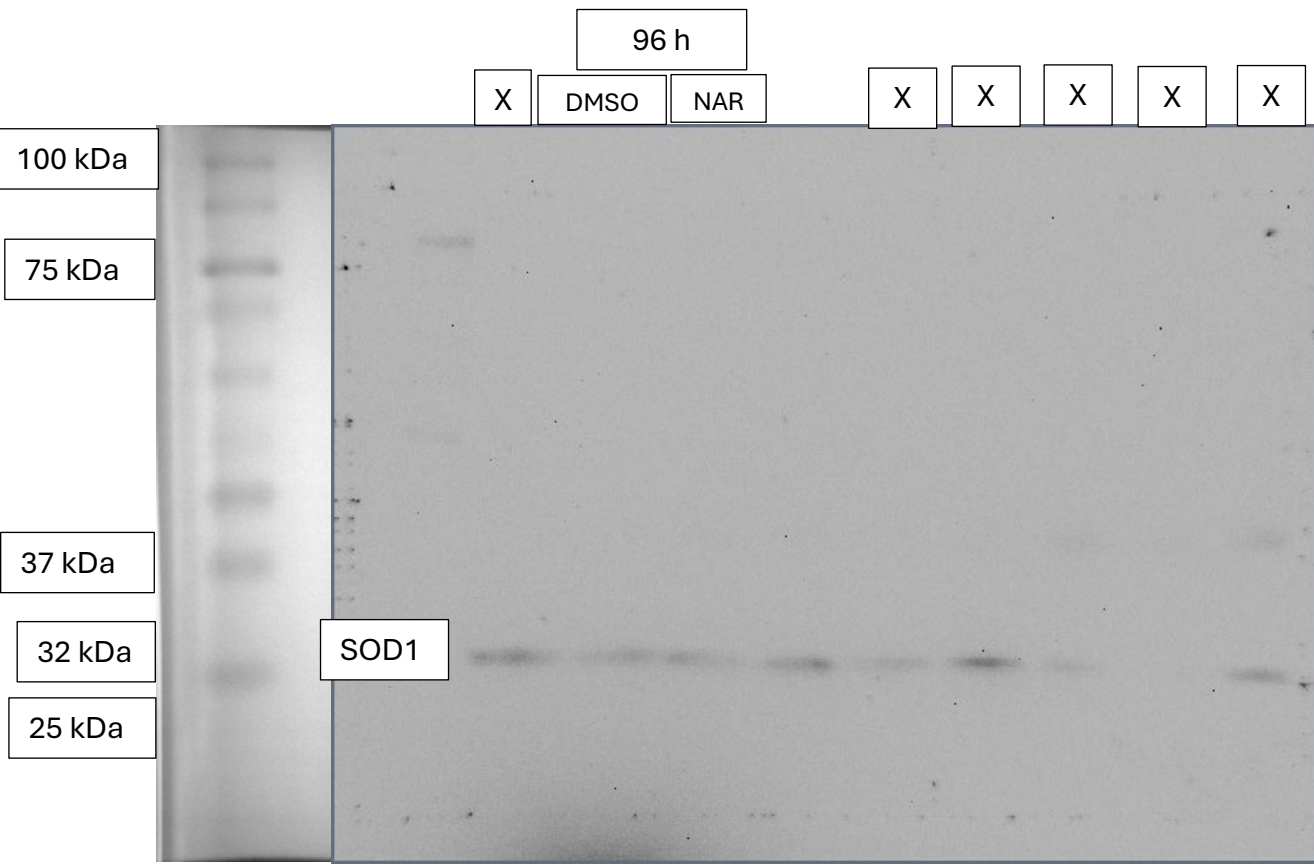

Supplement: S1 File — (PDF) [file pone.0320020.s005.pdf]
