# Supplementary material for: Naringenin impairs mitochondrial function via ROS to induce apoptosis in tamoxifen resistant MCF-7 breast cancer cells
Source: PLoS One. 2025 Apr 3;20(4):e0320020. doi: 10.1371/journal.pone.0320020 (PMC11967926; doi:10.1371/journal.pone.0320020)

S4 Fig. 7A

Trial 2

T2 20X Cont

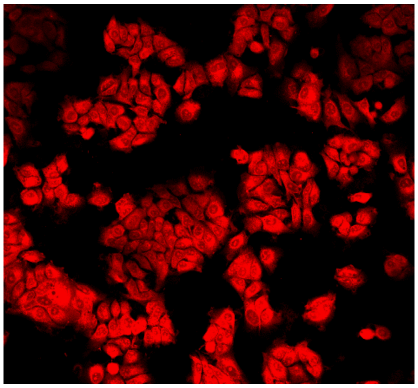

T2 40X Cont

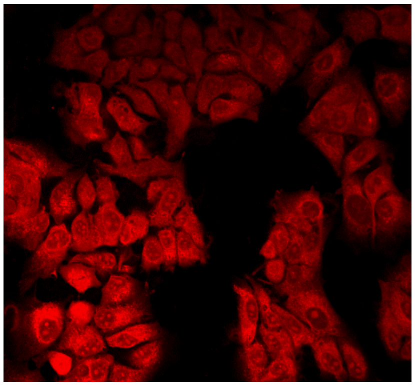

T2 20X DMSO

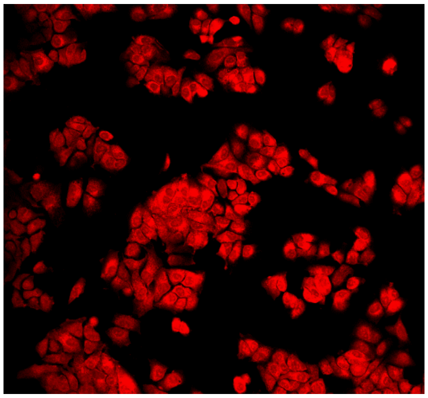

T2 40X DMSO

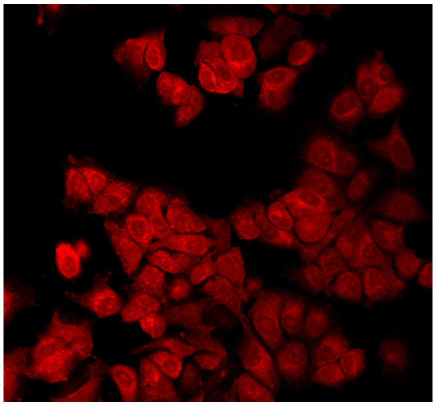

T2 20X NAR

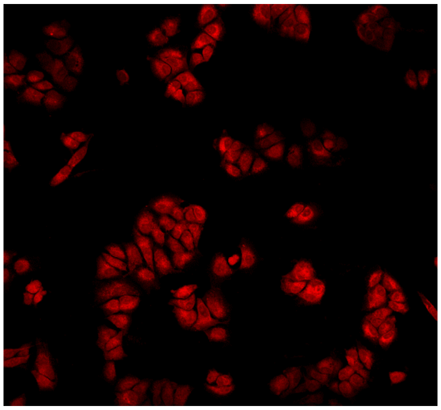

T2 40X NAR

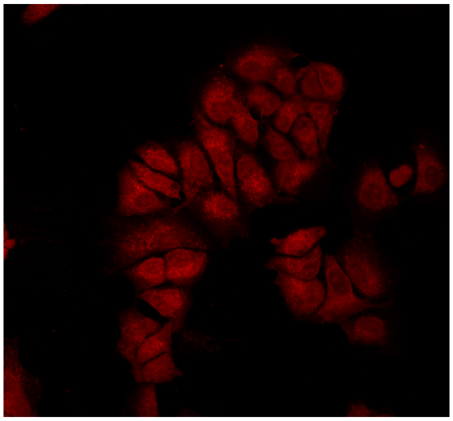

Trial 3

T3 20X Control

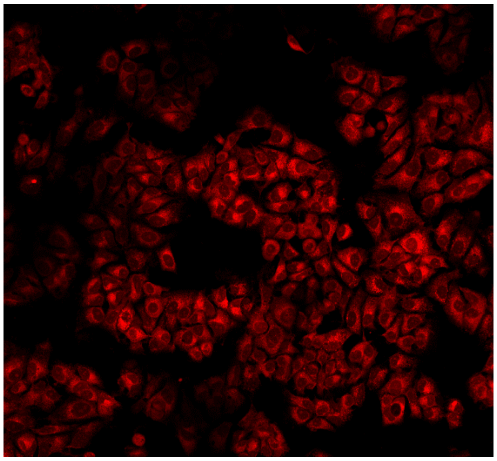

T3 40X Control

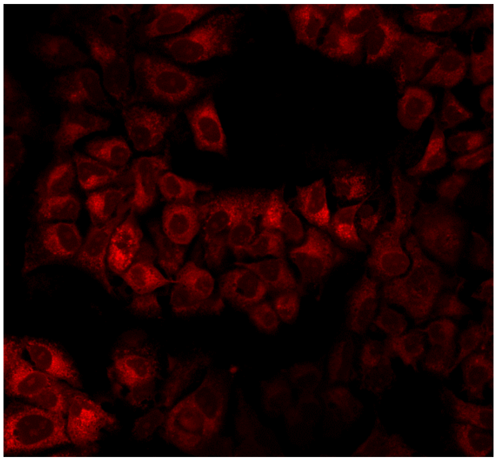

T3 20X DMSO

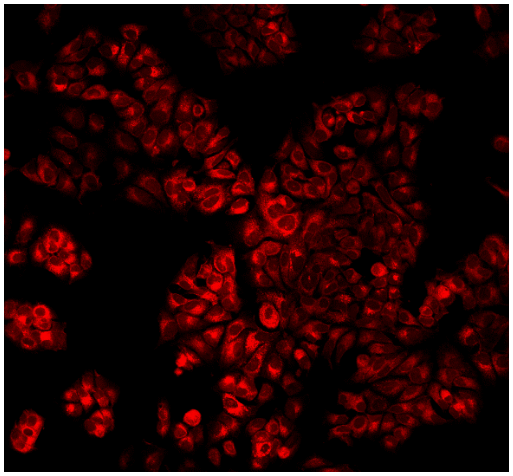

T3 40X DMSO

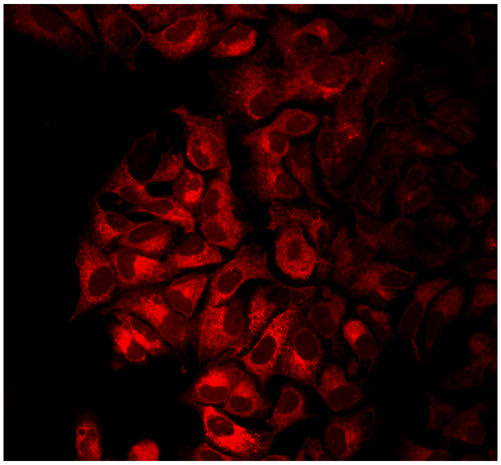

T3 20X NAR

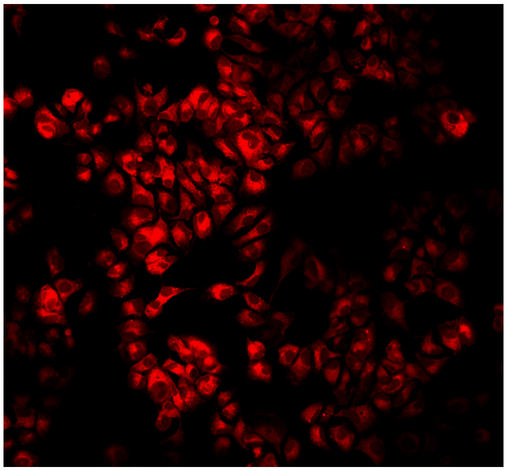

T3 40X NAR

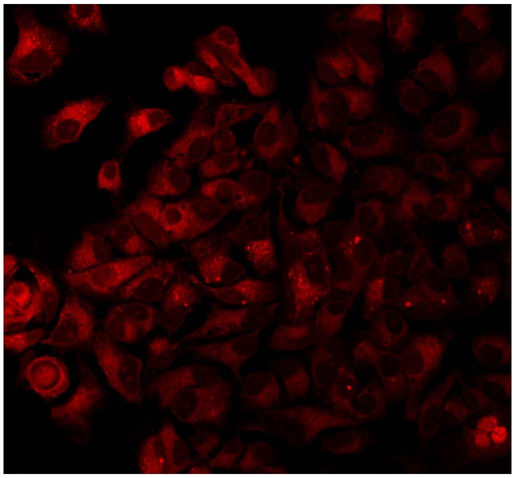

Supplement: S4 Fig — (PDF) [file pone.0320020.s004.pdf]
