# Supplementary material for: Naringenin impairs mitochondrial function via ROS to induce apoptosis in tamoxifen resistant MCF-7 breast cancer cells
Source: PLoS One. 2025 Apr 3;20(4):e0320020. doi: 10.1371/journal.pone.0320020 (PMC11967926; doi:10.1371/journal.pone.0320020)
Supplement: S2 File — (PDF) [file pone.0320020.s006.pdf]

Raw Data

S2. Figure 1 Raw Data

	**Trial 1**	**Trial 2**	**Trial 3**
**24 h**			
Control	150656.8	517441.7	357894.6
NAR	82895.93	575507.1	301007.6
**48 h**			
Control	113365.3	629434.2	384734.9
NAR	65262.6	498488.4	365057.2
**96 h**			
Control	104684	699713.9	2792742
NAR	87106.09	392108.3	925525.7

S2. Figure 2 Raw Data

Figure 2G					
**Annexin (+)**	**T1**	**T2**	**T3**	**Average**	**SEM**
**24 h DMSO**	5.8	7.5	7.6	6.966667	0.584047
**24 h NAR**	14.5	19.5	16.1	16.7	1.474223
**48 h DMSO**	5.7	8.2	10.7	8.2	1.443376
**48 h NAR**	24.5	18.4	25	22.63333	2.121582
**96 h DMSO**	7.6	6.3	9.8	7.9	1.021437
**96 h NAR**	18.9	18.4	22.7	20	1.357694

S2. Figure 3 Raw Data

Figure 3G							
	**T1**	**T2**	**T3**	**T4**			
**24 h**	**M1**	**M1**	**M1**	**M1**	**AVG**	**STDEV**	**SEM**
**Control**	49.58	30.91	32.69	21.8	33.745	5.841698	2.920849
**DMSO**	37.58	29.35	30.92	30.4	32.0625	3.735848	1.867924
**NAR**	37.7	60.52	38.13	28.1	41.1125	5.67077	2.835385
	**M2**	**M2**	**M2**	**M2**	**AVG**	**STDEV**	**SEM**
**Control**	21.4	28.33	35.09	31.8	29.155	5.860629	2.930314
**DMSO**	19.8	26.41	34.26	31.24	27.9275	6.309577	3.154788
**NAR**	18.4	22.54	15.14	31.58	18.69333333	7.119426	3.559713
	**M3**	**M3**	**M3**	**M3**	**AVG**	**STDEV**	**SEM**
**Control**	22.06	33.52	34.78	18.9	27.315	8.013645	4.006822
**DMSO**	35.79	35.22	37.63	14.5	30.785	10.90527	5.452633
**NAR**	39.76	24.34	28.8	19.9	28.2	8.520235	4.260117

Figure 3H							
	**T1**	**T2**	**T3**	**T4**			
**48 h**	**M1**	**M1**	**M1**	**M1**	**AVG**	**STDEV**	**SEM**
**Control**	44.25	42.86	44.65	33.5	41.315	5.266172	2.633086
**DMSO**	47.49	42.2	44.06	32.9	41.6625	5.979063	2.989532
**NAR**	50.29	53.88	52.84	37.1	52.33667	1.847169	1.066464
	**M2**	**M2**	**M2**	**M2**	**AVG**	**STDEV**	**SEM**
**Control**	20.59	29.42	24.43	17.2	22.91	5.249667	2.624833
**DMSO**	23.55	27.64	25.87	16.2	23.315	5.030311	2.515156
**NAR**	16.36	16.04	16.32	10.1	14.705	3.073299	1.536649
	**M3**	**M3**	**M3**	**M3**	**AVG**	**STDEV**	**SEM**
**Control**	35.16	27.26	28.35	17.9	27.1675	7.098673	3.549336
**DMSO**	28.96	29.56	27.95	17.2	25.9175	5.849515	2.924757
**NAR**	33.35	29.92	30.8	16.5	27.6425	7.569405	3.784702

Figure 3I							
	**T1**	**T2**	**T3**	**T4**			
**96 h**	**M1**	**M1**	**M1**	**M1**	**AVG**	**STDEV**	**SEM**
**Control**	45.88	51.61	50.5	30.1	49.33	3.038898	1.754508
**DMSO**	49.15	54.7	52.91	27.8	46.14	2.832672	1.635444
**NAR**	55.36	58.86	55.51	38.4	56.57667	1.978846	1.142488
	**M2**	**M2**	**M2**	**M2**	**AVG**	**STDEV**	**SEM**
**Control**	23.19	22.13	17.45	15	19.4425	3.87179	1.935895
**DMSO**	19.42	18.83	17.69	13.2	18.64667	0.879451	0.507751
**NAR**	18.81	11.59	9.94	12	13.085	3.919137	1.959568
	**M3**	**M3**	**M3**	**M3**	**AVG**	**STDEV**	**SEM**
**Control**	30.92	26.26	32.05	15.7	26.2325	7.455429	3.727715
**DMSO**	31.29	26.47	28.75	17.7	26.0525	5.906112	2.953056
**NAR**	25.82	27.55	34.56	20.4	27.0825	5.84184	2.92092

Figure 3 J				
**Trial 1 Beta Actin**	**Value**	**Trial 1 Cyclin D1**	**Value**	**Cyclin D1/Beta Actin**
**24 h DMSO**	68.895	**24 h DMSO**	0.542	0.007867
**24 h NAR**	45.621	**24 h NAR**	0.513	0.011245
**48 h DMSO**	77.357	**48 h DMSO**	0.397	0.005132
**48 h NAR**	58.196	**48 h NAR**	0.537	0.009227
**96 h DMSO**	29.742	**96 h DMSO**	0.397	0.013348
**96 h NAR**	41.68	**96 h NAR**	0.062	0.001488
**Trial 2 Beta Actin**	**Value**	**Trial 2 Cyclin D1**	**Value**	**Cyclin D1/Beta Actin**
**24 h DMSO**	55.727	**24 h DMSO**	0.188	0.003374
**24 h NAR**	83.187	**24 h NAR**	0.316	0.003799
**48 h DMSO**	87.861	**48 h DMSO**	0.62	0.007057
**48 h NAR**	85.864	**48 h NAR**	0.519	0.006044
**96 h DMSO**	88.082	**96 h DMSO**	0.598	0.006789
**96 h NAR**	44.934	**96 h NAR**	0.391	0.008702
**Trial 3 Beta Actin**	**Value**	**Trial 3 Cyclin D1**	**Value**	**Cyclin D1/Beta Actin**
**24 h DMSO**	83.671	**24 h DMSO**	15.331	0.18323
**24 h NAR**	79.851	**24 h NAR**	10.71	0.134125
**48 h DMSO**	81.479	**48 h DMSO**	20.53	0.251967
**48 h NAR**	75.74	**48 h NAR**	10.934	0.144362
**96 h DMSO**	77.239	**96 h DMSO**	15.06	0.194979
**96 h NAR**	37.517	**96 h NAR**	2.18	0.058107

S2. Figure 4 Raw Data

Figure 4D								
**Trial 1**	**% Shifted**	**Trial 2**	**% Shifted**	**Trial 3**	**% Shifted**	**AVG**	**STDEVA**	**SEM**
Control – dye	0	Control – dye	0.0405	Control – dye	0.1636	0.068033	0.085204	0.049193
Control + dye	24.9382	Control + dye	5.33549	Control + dye	25.3897	18.55446	11.45019	6.610772
2h DMSO	27.6777	2h DMSO	11.3655	2h DMSO	33.3403	24.12783	11.40939	6.587213
2h NAR	65.639	2h NAR	80.8642	2h NAR	81.6457	76.04963	9.024337	5.210203
3h DMSO	6.97581	3h DMSO	14.0984	3h DMSO	5.1308	8.735003	4.735561	2.734077
3h NAR	74.8308	3h NAR	78.4784	3h NAR	51.5256	68.27827	14.62242	8.442257
6h DMSO	7.06577	6h DMSO	28.8944	6h DMSO	13.2251	16.39509	11.25428	6.497664
6h NAR	88.0754	6h NAR	77.381	6h NAR	49.5128	71.6564	19.90846	11.49416

S2. Figure 5 Raw Data

Figure 5A

Trial 1	% shifted	Trial 2	% Shifted	Trial 3	% Shifted	Trial 4	% Shifted	AVG	STDEVA	SEM
Control - dye	0.025214	Control – dye	0.031289	Control – dye	0	Control – dye	0	0.014126	0.016499	0.009525
Control + dye	42.3948	Control + dye	47.2615	Control + dye	42.4358	Control + dye	50.997	45.77228	4.165531	2.40497
2h DMSO	49.5611	2h DMSO	51.9115	2h DMSO	53.9216	2h DMSO	42.8087	49.55073	4.835039	2.791511
2h NAR	84.5814	2h NAR	77.519	2h NAR	72.1473	2h NAR	93.7032	81.98773	9.3235	5.382925
3h no dye	0.027933	3h no dye	0	3h no dye	0.034904	3h no dye	0.020964	0.02095	0.015082	0.008707
3h control dye	60.2112	3h control dye	63.6965	3h control dye	50.6594	3h control dye	65.195	59.94053	6.530171	3.770196
3h DMSO	52.6098	3h DMSO	54.883	3h DMSO	62.3148	3h DMSO	55.2693	56.26923	4.197691	2.423538
3h NAR	88.5367	3h NAR	82.6679	3h NAR	87.7961	3h NAR	93.1849	88.0464	4.30641	2.486307
6h no dye	0.033014	6h no dye	0	6h no dye	0	6h no dye	0	0.008254	0.016507	0.00953
6h control dye	60.0554	6h control dye	66.9756	6h control dye	66.1228	6h control dye	66.8836	65.00935	3.324673	1.919501
6h DMSO	73.8926	6h DMSO	67.2976	6h DMSO	68.3138	6h DMSO	66.9237	67.5117	3.244064	1.872961
6h NAR	74.0847	6h NAR	72.6844	6h NAR	91.2402	6h NAR	88.6804	84.66843	9.643862	5.567886

Figure 5B

Trial 1	% shifted	Trial 2	% Shifted	Trial 3	% Shifted	AVG	STDEVA	SEM
Control - dye	1.9297	Control - dye	0.512055	Control – dye	0.784896	1.07555	0.75219	0.434277
Control + dye	59.5277	Control + dye	61.7232	Control + dye	53.089	58.1133	4.48751	2.590865
24h DMSO	53.9517	24h DMSO	66.186	24h DMSO	58.0591	59.39893	6.226226	3.594713
24h NAR	78.1747	24h NAR	78.0189	24h NAR	65.0146	73.73607	7.553413	4.360965
48h control dye	69.1414	48h control dye	65.94	48h control dye	56.7464	63.9426	6.434377	3.714889
48h DMSO	68.5981	48h DMSO	69.6	48h DMSO	60.4998	66.23263	4.989988	2.880971
48h NAR	78.1618	48h NAR	76.3424	48h NAR	77.5005	77.3349	0.920935	0.531702
96h control dye	59.6418	96h control dye	58.3819	96h control dye	50.1995	56.0744	5.126663	2.95988
96h DMSO	56.4023	96h DMSO	58.1268	96h DMSO	60.9881	58.50573	2.316265	1.337296
96h NAR	83.2155	96h NAR	77.4424	96h NAR	83.8954	81.51777	3.545705	2.047114

S2. Figure 6 Raw Data

Figure 6A-D					
**Trial 1**	**GXP1 Average C_T**	**SOD1 Average C_T**	**SOD2 Average C_T**	**Catalase Avg C_T**	**GAPDH Average C_T**
72h Control	20.64881	18.43249	23.10164	23.74796	19.4632
72h DMSO	21.12902	18.30405	22.96359	23.96074	19.29852
72h NAR	22.29869	18.52794	23.26393	24.02564	19.57908
96h Control	20.04249	18.48596	22.94146	24.33671	19.23985
96h DMSO	21.15146	18.57876	23.23327	24.57983	19.61716
96h NAR	22.96476	18.62954	23.61106	24.52131	19.96988
**Trial 2**	**GXP1 Average C_T**	**SOD1 Average C_T**	**SOD2 Average C_T**	**Catalase Avg C_T**	**GAPDH Average C_T**
72h Control	20.62398	20.44341946	21.61224	21.57541	12.84677
72h DMSO	20.3101	19.78042068	21.84328	21.05789566	12.29985
72h NAR	22.50204372	19.90736675	21.71116638	21.60270882	12.95539
96h Control	19.63836575	19.32165337	21.74047	21.83588	12.60987
96h DMSO	19.65383911	19.33631039	21.29894066	21.70947	12.20308
96h NAR	21.85672	19.86019325	21.89133072	21.85194397	13.17627
**Trial 3**	**GXP1 Average C_T**	**SOD1 Average C_T**	**SOD2 Average C_T**	**Catalase Avg C_T**	**GAPDH Average C_T**
72h Control	19.72571564	18.53953552	21.75683403	23.38781548	15.75302792
72h DMSO	19.66187763	18.09187889	21.69898796	23.02373695	15.50147152
72h NAR	21.36606789	17.74573231	22.33332253	23.57167149	15.80679035
96h Control	18.59887123	17.28638649	21.36276054	23.46258736	15.27282429
96h DMSO	18.76861954	17.19241333	21.42618561	23.41938972	15.13060093
96h NAR	21.58744717	17.74573231	22.39375305	23.63776245	15.86420441

Figure 6E					
**Set 1 (April Catalase)**	**Value**	**Set 2 (April Catalase)**	**Value**	**Set 3 (April Catalase)**	**Value**
96 DMSO	1.863	96 DMSO	2.753	96 DMSO	3.151
96 NAR	2.421	96 NAR	3.381	96 NAR	3.954
**Set 1 (May Catalase)**	**Value**	**Set 2 (May Catalase)**	**Value**	**Set 3 (May Catalase)**	**Value**
96 DMSO	0.605	96 DMSO	0.573	96 DMSO	0.608
96 NAR	0.053	96 NAR	0.069	96 NAR	0.089
**Set 1 (September Catalase)**	**Value**	**Set 2 (September Catalase)**	**Value**	**Set 3 (September Catalase)**	**Value**
96 DMSO	7.521	96 DMSO	10.732	96 DMSO	9.358
96 NAR	8.314	96 NAR	11.731	96 NAR	10.333
**Set 1 (April SOD1)**	**Value**	**Set 2 (April SOD1)**	**Value**	**Set 3 (April SOD1)**	**Value**
96 DMSO	0.45	96 DMSO	0.42	96 DMSO	0.43
96 NAR	0.392	96 NAR	0.293	96 NAR	0.331
**Set 1 (May SOD1)**	**Value**	**Set 2 (May SOD1)**	**Value**	**Set 3 (May SOD1)**	**Value**
96 DMSO	0.813	96 DMSO	0.932	96 DMSO	0.828
96 NAR	0.464	96 NAR	0.459	96 NAR	0.416
**Set 1 (September SOD1)**	**Value**	**Set 2 (September SOD1)**	**Value**	**Set 3 (September SOD1)**	**Value**
96 DMSO	0.529	96 DMSO	0.6	96 DMSO	0.65
96 NAR	0.822	96 NAR	1.029	96 NAR	1.153

S2. Figure 7 Raw Data

Figure 7B													
Average Intensity										Avg	Avg Treat.	STDEVA	SEM
Control.1	1724.70	2299.40	1621.90	1964.00	2351.40	1822.30	2323.10	2329.70	1694.30	2014.53	1996.58	282.3523	54.33873
Control.2	2074.10	1758.10	1673.00	2325.30	1917.50	1687.20	1571.70	2021.90	2250.20	1919.89			
Control.3	1971.70	2331.50	1641.60	2422.50	1851.10	2363.10	2151.60	2043.00	1721.80	2055.32			
DMSO.1	2155.30	2138.50	1495.70	2344.00	2320.80	2338.80	1617.90	1693.70	2434.50	2059.91	1990.24	319.2821	61.44587
DMSO.2	2277.20	1812.90	2039.80	1585.00	1568.80	2354.10	1675.10	2022.20	1685.00	1891.12			
DMSO.3	2031.50	1657.90	1632.70	2501.50	2483.10	2133.40	1946.60	1831.60	1958.80	2019.68			
Nar.1	938.35	1070.30	1284.20	877.38	1083.40	1023.90	959.68	1322.30	1327.80	1098.59	989.48	212.3059	40.85828
Nar.2	909.86	1259.50	1148.30	825.00	842.92	1023.50	1060.90	862.92	1252.60	1020.61			
Nar.3	731.00	692.57	910.57	1098.00	1235.90	600.32	813.76	979.68	581.32	849.24			
